# Genome-wide association study of fiber yield-related traits uncovers the novel genomic regions and candidate genes in Indian upland cotton (*Gossypium hirsutum* L.)

**DOI:** 10.3389/fpls.2023.1252746

**Published:** 2023-10-23

**Authors:** Babita Joshi, Sanjay Singh, Gopal Ji Tiwari, Harish Kumar, Narayanan Manikanda Boopathi, Sarika Jaiswal, Dibyendu Adhikari, Dinesh Kumar, Samir V. Sawant, Mir Asif Iquebal, Satya Narayan Jena

**Affiliations:** ^1^ Plant Genetic Resources and Improvement, CSIR-National Botanical Research Institute, Lucknow, India; ^2^ Academy of Scientific and Innovative Research (AcSIR), Ghaziabad, India; ^3^ Division of Agricultural Bioinformatics, ICAR-Indian Agricultural Statistics Research Institute, New Delhi, India; ^4^ Department of Plant Breeding and Genetics, Punjab Agricultural University, Regional Research Station, Faridkot, Punjab, India; ^5^ Department of Plant Biotechnology, Centre for Plant Molecular Biology and Biotechnology, Tamil Nadu Agricultural University, Coimbatore, Tamil Nadu, India; ^6^ Plant Ecology and Climate Change Science, CSIR-National Botanical Research Institute, Lucknow, India; ^7^ Molecular Biology & Biotechnology, CSIR-National Botanical Research Institute, Lucknow, India

**Keywords:** cotton, yield traits, GBS sequencing, SNP, GWAS, MLMM

## Abstract

Upland cotton (*Gossypium hirsutum L.*) is a major fiber crop that is cultivated worldwide and has significant economic importance. India harbors the largest area for cotton cultivation, but its fiber yield is still compromised and ranks 22nd in terms of productivity. Genetic improvement of cotton fiber yield traits is one of the major goals of cotton breeding, but the understanding of the genetic architecture underlying cotton fiber yield traits remains limited and unclear. To better decipher the genetic variation associated with fiber yield traits, we conducted a comprehensive genome-wide association mapping study using 117 Indian cotton germplasm for six yield-related traits. To accomplish this, we generated 2,41,086 high-quality single nucleotide polymorphism (SNP) markers using genotyping-by-sequencing (GBS) methods. Population structure, PCA, kinship, and phylogenetic analyses divided the germplasm into two sub-populations, showing weak relatedness among the germplasms. Through association analysis, 205 SNPs and 134 QTLs were identified to be significantly associated with the six fiber yield traits. In total, 39 novel QTLs were identified in the current study, whereas 95 QTLs overlapped with existing public domain data in a comparative analysis. Eight QTLs, *qGhBN_SCY_D6-1*, *qGhBN_SCY_D6-2*, *qGhBN_SCY_D6-*3, *qGhSI_LI_A5, qGhLI_SI_A13, qGhLI_SI_D9*, *qGhBW_SCY_A10*, and *qGhLP_BN_A8* were identified. Gene annotation of these fiber yield QTLs revealed 2,509 unique genes. These genes were predominantly enriched for different biological processes, such as plant cell wall synthesis, nutrient metabolism, and vegetative growth development in the gene ontology (GO) enrichment study. Furthermore, gene expression analysis using RNAseq data from 12 diverse cotton tissues identified 40 candidate genes (23 stable and 17 novel genes) to be transcriptionally active in different stages of fiber, ovule, and seed development. These findings have revealed a rich tapestry of genetic elements, including SNPs, QTLs, and candidate genes, and may have a high potential for improving fiber yield in future breeding programs for Indian cotton.

## 1 Introduction

Cotton is one of the most important cash crops used for natural textile fibers and oilseeds. It is cultivated worldwide as the primary raw material for the textile industry (Chen et al., 2007). It is derived from the Arabic word “quotn,” “gutum,” or “kutum” meaning those crops which possess spinnable fibers on their seed coat (Lee, 1984). Among the plant species commonly used by people, fiber-yielding crops hold the second position after food crops. Cotton serves as an excellent model for studying various aspects, such as plant cell elongation, evolution, cellulose biosynthesis, and plant polyploidization (Qin and Zhu, 2011; Huang et al., 2021). It belongs to the genus *Gossypium in the* family Malvaceae and is one of the largest genera in the Gossypieae tribe owing to its high species diversity. It encompasses approximately 44 diploid (2n = 2x = 26) and seven tetraploid (2n = 4x = 52) species (Grover et al., 2015; Ditta et al., 2018).

Cotton is cultivated in more than 123 countries, encompassing regions ranging from arid to semi-arid areas in the tropics and subtropics. Among the four cultivated species, upland cotton (*Gossypium hirsutum*) constitutes ~95% of the global cotton production because of its high adaptability to various environments and significant yield (Chen et al., 2007). Thus, compared with other cultivated species, most breeding and improvement programs related to fiber yield, fiber quality, insect resistance, and drought tolerance, are mainly focused on upland cotton. Genetic improvement for high cotton fiber yield has always remained the primary focus of cotton breeders to increase their productivity. With progress and advancement in the textile industry, the demand for high fiber yield and quality is growing exponentially. Although fiber quality is an essential trait in cotton breeding programs (Rong et al., 2007; Said et al., 2013), enhancing cotton fiber yield using Indian cotton varieties is still a primary goal. In comparison to the rest of the world, India has the largest land area under cotton cultivation (12,150 thousand hectares), yet its productivity is greatly compromised (only 457 kg/ha) by 2022 (US Department of Agriculture, https://usda.library.cornell.edu/). In India, most cotton cultivars are released using conventional breeding techniques based on morphological traits that are affected by agronomic practices and environmental changes. Consequently, the diversity and quality of elite cotton are reduced daily because of the narrow genetic background, domestication, and selection of elite cotton cultivars (Iqbal et al., 2001; Rungis et al., 2005; Abdurakhmonov et al., 2012); thus, genetic improvement in cotton fiber yield and other quality traits is still challenging.

Cotton fiber yield is a multifaceted quantitative trait influenced by various traits, including boll weight (BW), boll number (BN), seed cotton weight (SCW), lint percentage (LP), lint index (LI), seed index (SI), first fruit branch position (FFBP), plant height (PH), flowering period (FP), fruit spur branch number (FSBN), and many others (Li F. et al., 2018; Sun et al., 2018). Such quantitative traits are governed by both quantitative trait loci (QTLs) and environmental factors, which are difficult to concurrently improve using traditional breeding techniques. However, the advancement of applied genomics research has introduced the use of QTL-linked or QTL-associated molecular markers in marker-assisted selection (MAS) and/or genomic selection programs. These emerging techniques offer promising avenues for enhancing the efficiency of cotton breeding and simultaneously targeting specific traits. QTL mapping has been extensively employed to analyze the genetic variations underlying complex traits in cotton, including fiber quality and yield component traits (Rong et al., 2007; Said et al., 2013; Said et al., 2015). Over the last 20 years, biparental linkage mapping in upland cotton has led to the identification of numerous QTLs associated with cotton yield (Zhang et al., 2005; Abdurakhmonov et al., 2007; Shen et al., 2007; Wan et al., 2007; Liu et al., 2012; Yu et al., 2013a; Yu et al., 2013b; Wang et al., 2014; Liu D. et al., 2015). Nevertheless, biparental QTL mapping encounters two main challenges: limited allelic diversity and restricted genomic resolution due to the relatively low number of recombination events that occur throughout the development of the mapping population (Jannink et al., 2001; Flint-Garcia et al., 2005; Hall et al., 2010).

To overcome these limitations of bi-parental QTL mapping, association mapping offers an alternative approach for mapping QTL, which relies on linkage disequilibrium (LD). It determines whether particular alleles in a population are more frequently associated with specific phenotypes than expected, thereby providing insights into the genetic basis of complex traits (Flint-Garcia et al., 2003). In addition, this analysis can be applied to a large natural population, allowing the identification of traces of genetic crossovers and loci responsible for traits at a much higher resolution than previously reported techniques. In recent years, genome-wide association studies (GWASs) have emerged as a more precise and cost-effective method for identifying significant QTLs or genes linked to complex traits than linkage mapping. The first association mapping study in cotton focusing on fiber quality was reported in *G. arboretum* (Kantartzi and Stewart, 2008). Subsequently, association mapping has been extensively applied in upland cotton to investigate crucial economic traits, including fiber quality (Abdurakhmonov et al., 2008; Nie et al., 2016; Liu et al., 2020; Song et al., 2021), seed oil content (Liu G. et al., 2015; Yuan et al., 2018; Zhao et al., 2019), fiber yield (Ademe et al., 2017; Sun et al., 2018; GUO et al., 2021; Niu et al., 2023), and biotic and abiotic stresses (Fang et al., 2010; Bardak et al., 2021; Xu et al., 2021; Zhang et al., 2021; Zhao et al., 2021), as well as those associated with epistasis and environmental interactions (Jia et al., 2014).

To date, studies on association mapping using Indian germplasm are scarce and were first reported by Handi et al. (2017), who used a cotton 63 K SNP chip to associate fiber yield and fiber quality traits in 201 upland cotton germplasm lines. Owing to their limitations, SNP chip-based studies only fetch SNPs at a specific location in the genome (where relevant information is gathered), and genotyping-by-sequencing (GBS) methods, on the other hand, proved to be an attractive approach for discovering and genotyping high-density SNPs. Although other marker classes can also be used for this study (for example, Kumar et al. (2021) used SSRs), SNPs demonstrated higher resolution in revealing genetic relatedness as well as delineating population structure in crops. Additionally, since SNP markers are abundant and primarily derived from genes, genetic diversity studies using these markers can reveal the functional variation that can be used in association mapping studies for specific traits (Singh et al., 2013).

Therefore, the specific goals of the present study were to (a) examine the phenotypic variability within the upland cotton germplasm released from different cotton growing belts in India; (b) explore the QTNs (qualitative trait nucleotides) or SNP markers underlying fiber yield traits; (c) compare identified QTLs with previously reported QTLs for yield traits, if available; and (d) identify and validate through expressional profiling of putative candidate genes found within the genomic regions controlling yield traits. Our results using 117 diverse Indian germplasms and 2,41,086 SNPs identified many stable and novel QTL/genes that may offer crucial information on the genetic control of fiber yield traits in cotton. This information will aid in improving cotton yield and the development of elite Indian cotton varieties through marker-assisted breeding programs.

## 2 Materials and methods

### 2.1 Plant material

An association panel consisting of 117 Indian upland cotton (*G. hirsutum* L.) germplasm was procured from All India Coordinated Research Project (AICRP) on Cotton, ICAR-Central Institute for Cotton Research, Regional Station, Coimbatore, India. These materials were selected based on their phenotypic expression with reference to fiber yield. The germplasms used in this study constitute the varieties released from diverse cotton-growing belts (Northern, Southern, and Central) of India. Out these, 31, 43, and 32 germplasms were from the northern, southern, and central zones, respectively, and information was unavailable for 11 germplasms zones (**Supplementary Table S1**).

### 2.2 Field experiment locations

Field experiments for precise phenotyping were conducted in two different natural environments in India’s cotton-growing regions: the northern and southern zones. In the northern zone, the investigated plant materials were evaluated at the Punjab Agriculture University, Regional Research Station, Faridkot (30°40’32.4”N 74°44’57.3” E; hereafter referred as E1) and in the southern zone, it was grown at Tamil Nadu Agriculture University, Coimbatore (11°07’3.36”N 76°59’39.91” E; hereafter referred as E2). There was a significant disparity in the agro-climatic conditions between the two cotton-growing regions, including variations in the soil type, rainfall, temperature, and growing season. Cotton cultivation in E1 was done in alluvial soil during May–November 2021 (under high temperatures), while in E2, plant materials were grown under red soil at relatively lower temperatures.

### 2.3 Experimental design and phenotypic trait measurements

The plants comprising the association panels were sown following a randomized block design (RBD) with ten biological replicates of each germplasm accession. The plant-to-plant spacing was 45 cm, whereas the row-to-row spacing was 90 cm, and there were 10 plants in each row.

The cotton crop in E1 was established following regular practices such as field preparation with fine tilth, sowing with a single seed/hill, fertilizer applications (basal: 150 kg of urea and 50 kg muriate of potash, and top dressing: 50 kg urea and 25 kg diammonium phosphate). Imidacloprid was applied to control mites and sucking pest infestation that was noticed during the early period of the cropping program (25th DAS). In E2, cotton cultivation was supplemented with 12 kg/acre of phosphorus as a pre-planting application, followed by separate applications of 15 kg/acre of nitrogen during both the thinning and flowering stages. Osheen 20 SG (dinotefuran) was applied to safeguard the cotton crop against sucking pests such as whiteflies and jassids to maintain healthy crops.

At maturity, all opened bolls were harvested from the surviving healthy individual plants to estimate the fiber yield traits, such as BN, BW(g), LI (g), LP (%), SCY(g/plant), and SI (g). For BN, the total number of bolls was counted from each plant, BW was measured as the average weight of 10 mature healthy bolls from each plant, and SCY per plant was the total weight of the seed along with the lint. Other traits were subsequently measured after ginning the cotton bolls; LP (Ginning Out Turn) is defined as the percentage of lint weight obtained from a given weight of seed cotton and was calculated using the formula given below; LI is the weight of the lint produced by the 100 seeds and it was calculated using the formula given below; SI was measured as the weight of 100 healthy seeds.


$$\text{Lint}\%(\text{GOT})=\frac{\text{Lint weight in a sample}}{\text{Seed cotton weight}}\times 100$$

$$\text{Lint Index}(\text{LI})=\frac{\text{SI}\times \text{Lint}\%}{100-\text{Lint}\%}$$

### 2.4 Phenotypic data analysis

The mean, coefficient of variation (CV), standard deviation (SD), Pearson’s linear correlation coefficients, variance components, Broad sense heritability (h^2^), and BLUP-based prediction of the mean for the multi-environment trait (two locations) were calculated using the “METAN,” “Phenotype,” and “corrplot” packages in R environment (Team, 2016). GWAS analysis was performed using the individual mean of the two locations and the BLUPed mean of two location trait data of 117 germplasms.

### 2.5 Genomic DNA extraction, GBS library preparation, genotyping, and SNP call

Genomic DNA was extracted from fresh young leaves using a modified cetyltrimethylammonium bromide (CTAB) method (Shukla et al., 2021). To ensure the accuracy and reliability of the DNA samples, stringent quality control (QC) procedures were performed using agarose gel electrophoresis, NanoDrop® 2000 spectrophotometer, and Qubit® 2.0 fluorometer. For library preparation, 0.3–0.6 0μg of high-quality genomic DNA was digested completely with the in *silico* optimized restriction enzyme set MseI (frequent cutter) and HaeIII_MspI (rare cutter) followed by efficient adapter ligation. After library preparation, high-throughput paired-end DNA sequencing was performed using the Illumina® HiSeq 2500 platform. Variant calling was performed using the *G*enome *A*nalysis *T*ool*k*it (GATK package, version 4.2.6.1) (Van der Auwera et al., 2013). In brief, the fastq files were converted into uBAM (unmapped BAM) format, followed by Marking of Illumina adapter sequence with the MarkIlluminaAdapters function. The marked uBAM files were converted back to fastq format and aligned to the *G. hirsutum* TM-1 reference genome (https://www.ncbi.nlm.nih.gov/assembly/GCF_007990345.1/) using BWA-mem, and then a clean BAM file was created using MergeBamAlignment (Li, 2013). Clean BAM files were sorted using Picard Sort Sam and marked for duplicate reads using a mark-duplicate function. Single genotype variant identification was performed with Haplotype Caller, and 117 VCF generated was used to create a variant database using GenomicsDBImport, followed by joint genotyping of 117 genotypes. The variants were filtered for QD< 2.0, FS > 60.0, MQ< 40.0, MQRankSum< −12.5, ReadPosRankSum< −8.0, SOR > 3.0, indel, minor allele frequency >0.05 and max-missing 0.1 for SNP trait association analysis (Van der Auwera and O’Connor, 2020).

### 2.6 Population structure, PCA, and genetic diversity analysis

To estimate the genetic differences between these 117 Indian cotton germplasms, population structure, molecular phylogenetic (neighbor-joining), principal component analysis (PCA), and kinship (k) analysis were performed. The filtered variants were pruned based on linkage disequilibrium (indep 50 5 0.5) using Plink (version 1.9) for PCA and population structure analysis (Purcell et al., 2007). The pruned variants were converted to a structure format using PGDSpider_2.1.1.5 (Lischer and Excoffier, 2012). STRUCTURE software (version 2.3.1) (Falush et al., 2007) was used to investigate the presence of subgroups in our association panel using Bayesian clustering. The Structure parameters used were 1–10 k, 10 replicates at each k, 100,000 burn-in, and 100,000 MCMC reps after burn-in, after which, the structure harvester was used to calculate the delta k value and prepare the Clumpp individual file. Clumpp was used to calculate the consensus membership coefficient value from the 10 replicates of the k run, and the inferred membership from Clumpp was used for further analysis and cluster visualization (Earl and VonHoldt, 2012). These links were used for principal component analysis (PCA). TASSEL5 was used for tree construction using the neighbor-joining method based on a modified Euclidean distance matrix (Bradbury et al., 2007).

### 2.7 SNP trait association analysis

The Genome Association and Prediction Integrated Tool (GAPIT), an R package was used for SNP (marker) trait association analysis. Three single-locus models (GLM, MLM, and CMLM), and three multi-locus models (MLMM, FarmCPU, and BLINK) were used (Wang and Zhang, 2021). The kinship coefficient matrix calculated in TASSEL5 was used as co-variables in the GWAS model and the PCA based on the Bayesian Information Content parameter implemented in GAPIT was used to account for population stratification for each trait with setting “model.selection=TRUE” to reduce the false discovery. The Manhattan and QQ plots were drawn using a significance threshold of p<0.000031 (−logP >4.5) using the ‘CMplot’ R package.

### 2.8 Identification of trait-associated QTLs and annotation of candidate genes

Following previously reported methods (Song et al., 2019; Sun Z. et al., 2017), LD sizes of ±200 kb upstream and downstream regions of significant SNPs were defined as QTLs, and SNPs within these regions were of the same locus. The co-location study of our GWAS-identified loci and previously reported results was implemented using the following steps: (1) all the previously reported QTLs and GWAS signals for yield-related traits were obtained from the http://cotton.zju.edu.cn/Qtl_phe.html database and association mapping reports; (2) the physical location/genomic coordinates of the SNPs in the QTL loci were retrieved through BLASTn (250 Bp flanks) against the *G. hirsutum* genome (Zhang et al., 2015; Hu et al., 2019); and (3) the coordinates of previous QTLs were compared with the QTLs identified in this study. Haplotype analysis was performed using the geneHapR package (Zhang et al., 2023), where haplogroups detected in five or more germplasms were considered for association analysis. The phenotypic value of each haplotype was assessed by calculating the average phenotypic value across the germplasm with each type of SNP locus linked to a specific target trait. In this study, favorable haplogroups were defined as the haplotype (combination of SNPs) that showed the highest average values over the other haplotypes. The gene located within the identified QTL region was mined and functional annotation was performed using BLAST2GO. Gene ontology enrichment analysis of the identified genes for individual traits was performed using the clusterProfiler package in R (Wu et al., 2021).

### 2.9 Expression analysis of genes in the associated region

RNA-Seq datasets obtained from various cotton tissues, including root, stem, leaf, torus, seed, cotyledon, ovule, fiber at 5 days post-anthesis (DPA), fiber at 10 DPA, fiber at 20 DPA, and fiber at 25 DPA, were downloaded from NCBI BioProject with the accession number PRJNA248163.The data were preprocessed for quality and adapter trimming using the Trimmomatic tool in the PE mode (Bolger et al., 2014). Alignment with the cotton genome was performed using the splice-aware aligner Hisat2. StringTie was used for count gene-level fragments per kilobase per million mapped read (FPKM) (Pertea et al., 2016). The FPKM count was normalized to zFPKM transformation with the zFPKM package of R, and genes with zFPKM values of ±3 in at least one sample were considered as expressed genes (Hart et al., 2013). The R package “pheatmap” (Kolde, 2019) was used to generate heat maps depicting the expression patterns of potential candidate genes.

## 3 Results

### 3.1 Phenotypic variability

The present study evaluated the phenotypic variability of six yield-related traits for an association panel of 117 Indian upland cotton germplasms in two environments, E1 and E2. A significant and extensive range of phenotypic variation was observed for all investigated traits (**Table 1**). BN, BW, LI, LP, SCY, and SI, exhibit values ranging from 17–39.2 (per plant), 2.24–5 (g), 3.44–5.89 (g), 31.2–46.2 (%), 45.8–177 (g/plant), and 5.55–10.3 (g), with an average of 24.6 g, 3.56 g, 4.54 g, 36.2% 88.1 (g/plant), and 7.87 g in E1, while for E2, all the six traits ranged from 15.3–30 (per plant), 2.12–4.8 (g), 3.4–6.53 (g), 32–44.3 (%), 42–120 (g/plant), and 5.65–10.8 (g), with an average of 22.9 g, 3.03 g, 4.52 g, 37.6%, 69.6 (g/plant), and 7.35 g, respectively.

**Table 1:** Description of phenotypic traits in two environments (E1 and E2).

Traits	Environment	Maximum	Minimum	Average	STDEV	CV (%)	Skewness	Kurtosis	Heritability (h2)
BN (per plant)	E1	39.2	17	24.6	4.01	16.3	0.507	-0.145	0.878
	E2	30	15.3	22.9	3.37	14.7	0.591	-0.476	0.783
BW (g)	E1	5	2.24	3.56	0.477	13.4	0.028	0.941	0.863
	E2	4.8	2.12	3.03	0.426	14.1	0.725	2.365	0.69
LI (%)	E1	5.89	3.44	4.54	0.443	9.76	0.116	1.057	0.748
	E2	6.53	3.4	4.52	0.576	12.7	0.296	2.044	0.643
LP (g)	E1	46.2	31.2	36.2	2.29	6.32	0.658	0.007	0.906
	E2	44.3	32	37.6	2.32	6.17	2.964	0.089	0.711
SCY (g/plant)	E1	177	45.8	88.1	21.8	24.7	0.955	0.729	0.696
	E2	120	42	69.6	14	20.1	2.183	1.18	0.466
SI (g)	E1	10.3	5.55	7.87	0.954	12.1	0.277	0.981	0.633
	E2	10.8	5.65	7.35	0.992	13.5	-0.201	1.401	0.515

**Figure 1:**
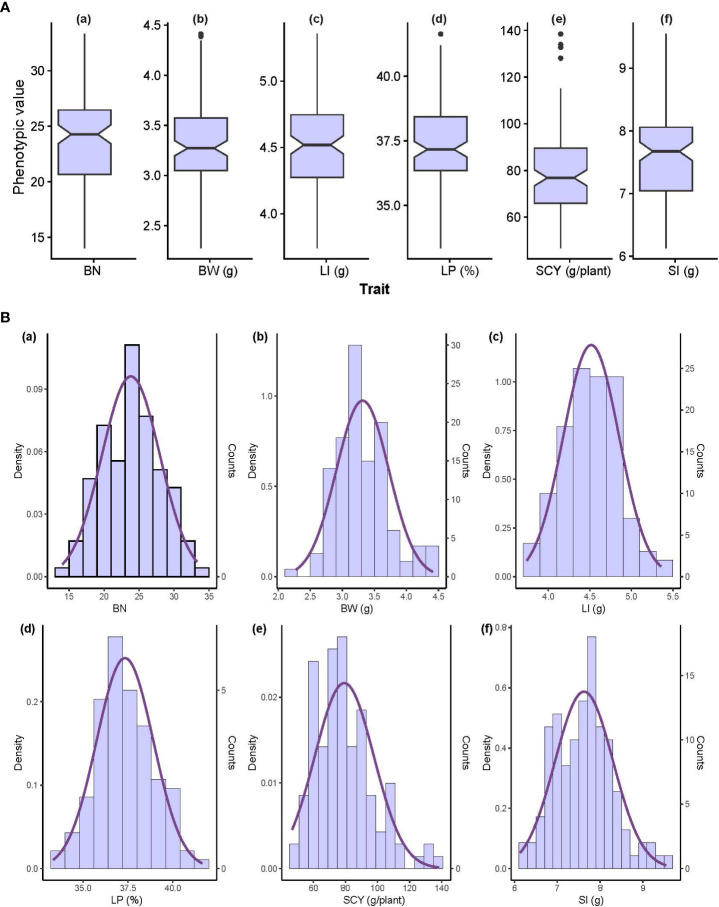
Phenotypic value distribution of 117 individual cotton genotypes using BLUP breeding values. **(A)** Box plot and **(B)** frequency distribution of six yield-related traits; the x-axis has trait labels and the y-axis contains the phenotypic value of each trait. BN, Boll Number; BW, Boll Weight; LI, Lint Index; LP, Lint Percentage; SCY, Seed Cotton Yield; SI, Seed Index.

**Figure 2:**
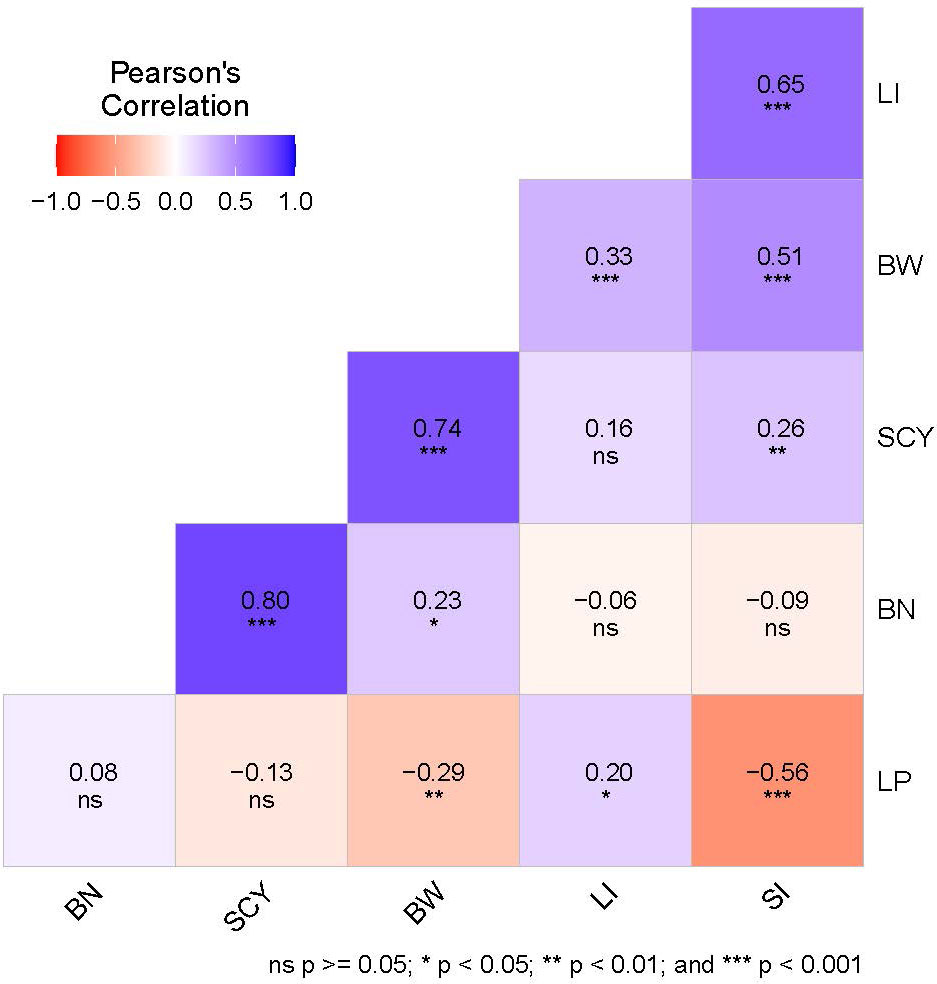
Correlation analysis of six yield-related traits. The color legend at the top shows the Pearson’s correlation coefficient value, and at the bottom, the significance level based on the p-value is given in which ns represents non-significant. BN, Boll Number; BW, Boll Weight; LI, Lint Index; LP, Lint Percentage; SCY, Seed Cotton Yield; SI, Seed Index.

The coefficient of variance (CV) and heritability (h^2^) ranged from 6.32% to 24.7% and 63.3% to 90.6%, respectively for E1, whereas in E2 the same was ranging from 6.17% to 20.1% and 46.6% to 78.3%, respectively for all six yield-related traits (**Table 1**). When compared with E1, E2 had lower heritability for all traits, indicating that the environmental conditions of E2 critically influence all six traits. The highest heritability was exhibited for SCY (90.6%) in E1, whereas the lowest was exhibited for LP (46.6%) in E2. The distribution pattern, box plot and phenotypic correlation analysis for yield-contributing traits were also calculated using the mean data of both environments as well as the data obtained from the BLUP analysis. Box plots and frequency distribution plots were drawn using BLUPed data (**Figures 1A, B**) and the trait mean data from two environments, E1 and E2 (**Supplementary Figures S1, S2**) to better understand the distribution pattern of the phenotypic data. All traits exhibited an approximately normal distribution pattern indicating that they were quantitative traits influenced by multiple genes. The correlation analysis among traits of the BLUP data (**Figure 2**) showed that SCY had a strong positive correlation with BN (0.80) and BW (0.74), a moderate positive correlation with SI (0.26), a weak positive correlation with LI (0.16), and a weak negative correlation with LP (−0.13). In addition, LP exhibited the highest negative correlation with SI (−0.56) and BW (−0.29). Moreover, a similar trend of correlation pattern was observed with the trait data obtained from E1, E2, and BLUPed-treated data (**Supplementary Table S2**).

The effects of genotype (G), environment (E), and genotype-environment interaction (G × E) for all six traits were assessed using analysis of variance (ANOVA). Significant variation (P<0.001) was observed, indicating that adequate variability was present for all six quantitative traits and was controlled by G, E, and G × E effects (**Supplementary Table S3**). Thus, the phenotypic data showed significant variation among the Indian cotton germplasms for yield-related traits, some of which were strongly correlated with others and were suitable for association mapping studies.

### 3.2 SNP genotyping and its data analysis

The GBS library constructed from 117 upland cotton germplasm, generated a vast amount of data of ~1,003.22 million pair-end reads (approximately 144.46 GB data, equates to an average of 8.574 million reads per sample) using Illumina® HiSeq 2500 platform. Details of the sequence read statistics are provided in **Supplementary Table S4**.

**Figure 3:**
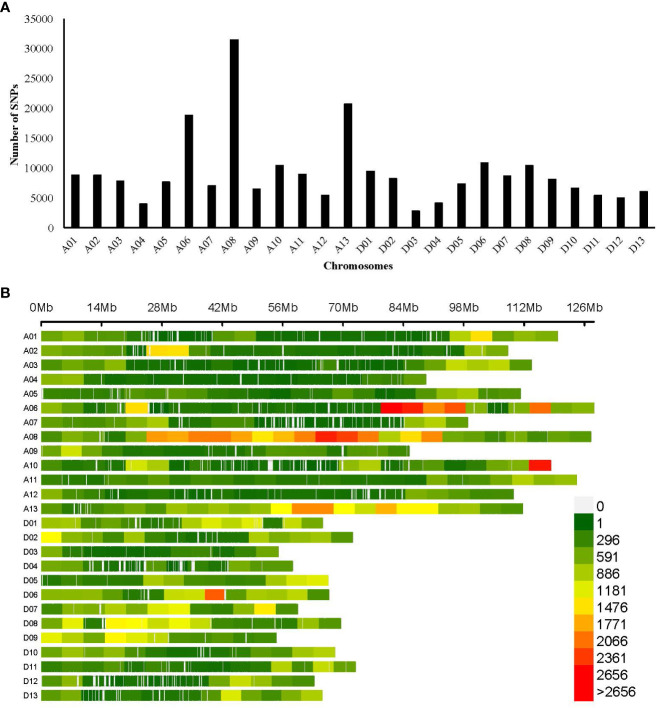
Genomic distribution of SNPs **(A)** Bar plot showing the total number of SNPs in 26 chromosomes of cotton; **(B)** Density plot of SNPs in the cotton genome, the horizontal bar indicates chromosomal length, and the color depicts the SNP density in the 5-Mb window. The color legend represents SNP density.

The SNP call performed with the GATK pipeline resulted in a total of 14,46,969 SNPs, of which 2,41,086 high-quality SNPs were retained after filtering (heterogeneity<0.3, missing<0.1, and MAF >0.05), which was used further for genetic variation and GWAS analysis. SNPs had transitions (A/G or C/T) of 5,549,405 bp and transversions (A/C, A/T, C/G, or G/T) of 2,339,098 bp, with a Ts/Tv ratio of 2.37%. The SNP distribution across the genome was not evenly distributed, depending on the genome content and gene density, with 1,47,347 and 93,739 SNPs in the At and Dt sub-genomes, respectively. The number of markers varied among the chromosomes, with a maximum number of SNPs (31,550) in Chr A08 and a minimum number of SNPs (2,860) found in Chr D03. The average SNP density throughout the genome was approximately one SNP per 11.36 kb (**Table 2**, **Figure 3A**). The density plot of SNPs for the 5 Mb region is represented as a heat map in **Figure 3B**.

**Table 2:** Chromosome-wise distribution of SNPs.

Chr	Chr Length (bp)	Number of SNPs (bp)	SNP density (kb/SNP)
A01	119761559	8916	13.43220716
A02	108141443	8933	12.10583712
A03	113693209	7826	14.52762701
A04	89180822	4041	22.06899827
A05	111098753	7703	14.42279021
A06	128195338	18852	6.800092192
A07	98902531	7089	13.95154902
A08	127495948	31550	4.041076006
A09	85335976	6509	13.11045875
A10	118182687	10527	11.22662553
A11	124181751	9012	13.77959953
A12	109474314	5549	19.72865633
A13	111646624	20840	5.357323608
D01	65205008	9523	6.847107844
D02	72186496	8343	8.652342802
D03	54956272	2860	19.21547972
D04	58229188	4128	14.10590795
D05	66484719	7321	9.081371261
D06	66684206	10934	6.098793305
D07	59440927	8758	6.787043503
D08	69427147	10410	6.669274448
D09	54445796	8158	6.673914685
D10	68089194	6633	10.26521845
D11	72823778	5526	13.17838907
D12	63255146	5012	12.62073943
D13	65099798	6133	10.61467438
Whole Genome	2281618630	241086	11.36011914

### 3.3 Population structure and kinship

**Figure 4:**
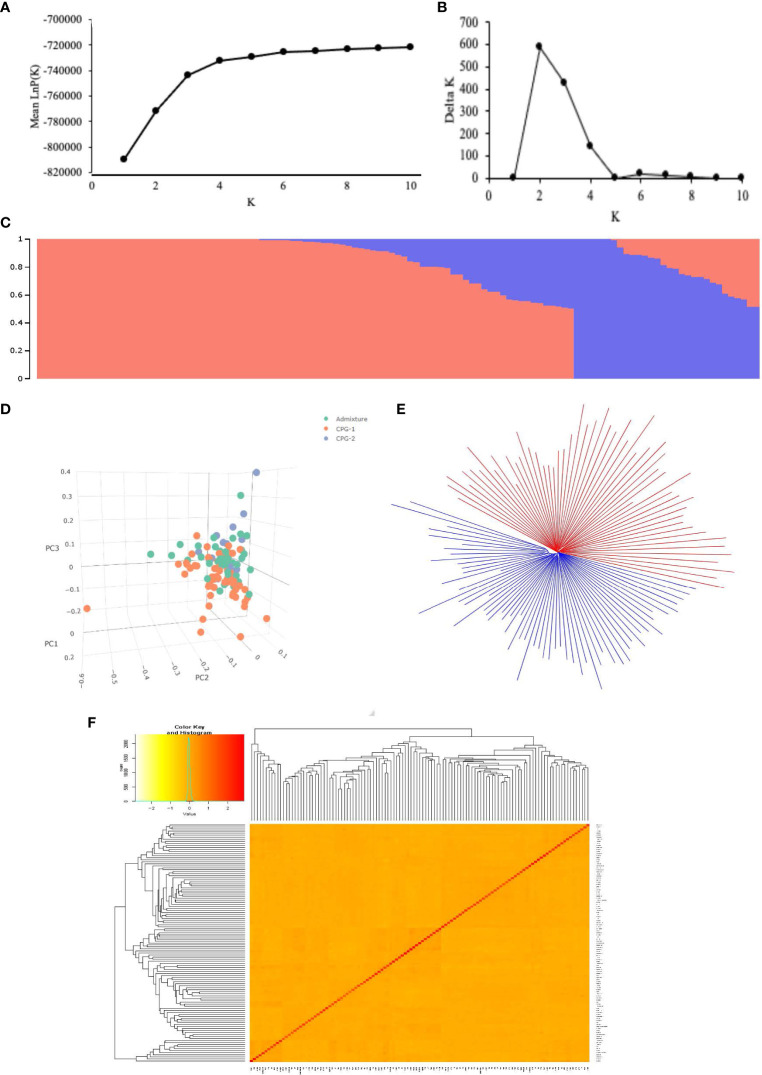
Population structure of the 117 cotton germplasms. **(A)** Mean LnP(K) values plotted from 1 to 10; **(B)** Ln(ΔK) values plotted from 1 to 10, for each value of K ten independent run was considered; **(C)** Population structure based on STRUCTURE where K = 2, the colored subsection within vertical bar represents membership coefficient value; **(D)** Plot of Principal component analysis showing genetic variation in cotton genotypes using first three principal component, the color label is based on STRUCTURE derived membership coefficient. Genotypes with ≥80% membership coefficient value were labeled as CG1 and CG2, the other were labeled as admixture; **(E)** NJ tree based on Nei’s genetic distances, the whole population was divided into two clusters; **(F)** Heatmap of Kinship coefficient matrix revealing the relationship between 117 individual Indian cotton genotypes.

This study employed multiple approaches to analyze the population structure, including Bayesian clustering using STRUCTURE software, principal component analysis (PCA), neighbor-joining (NJ) phylogenetic analysis, and kinship coefficient analysis. Understanding population structure is crucial in genome-wide association studies (GWAS) because the presence of a structure can influence the reliability of the association found. The LnP(K) value continuously increased from K = 1 to 10 (**Figure 4A**) with no inflection point in this panel. Moreover, Evanno’s ΔK showed a sharp peak at K = 2 (**Figure 4B**), suggesting that our population was divided into two subgroups (**Figure 4C**) designated as CPG1 and CPG2. CPG1 contained 65 and CPG2 contained 13 genotypes of cotton while 39 genotypes could not match the membership probability cut-off (0.8) of any cluster and were considered an admixture. CPG-1 consists of 12 (central), 20 (northern), 28 (southern), and five (unknown) genotypes, whereas CPG-2 consists of seven (central), one (northern), four (southern), and one (unknown), with an admixture containing 13 (central), 10 (northern), 11 (southern), and five (unknown) genotypes (**Supplementary Table S5**).

The STRUCTURE results were substantiated by principal component analysis (**Figure 4D**), a neighbor-joining (NJ) tree (**Figure 4E**), and a kinship matrix (**Figure 4F**). The first two principal components accounted for 14.34% of the total genetic variation, with PC1 and PC2 explaining 7.75% and 6.59%, respectively. The neighbor-joining (NJ) tree based on Nei’s genetic distances also demonstrated a division of the population into two main clusters, consistent with the STRUCTURE analysis. In addition, the kinship relatedness matrix showed two distinct subpopulations among 117 germplasms in this population. The kinship matrix revealed a relatively low degree of genetic relatedness, as indicated by the average pairwise relative kinship coefficient of 0.049. Among the cotton genotypes, most pairs (63.56%) exhibited estimated kinship values of zero. Approximately 31.05% of the kinship values ranged from 0 to 0.1, 3.66% ranged from 0.1 to 0.3, and the remaining pairs of genotypes (1.72%) displayed kinship values exceeding 0.3 (**Supplementary Figure S3**). This result indicates that the 117 Indian cotton germplasms are distantly and weakly related.

### 3.4 Genome-wide association mapping of fiber yield traits

**Figure 5:**
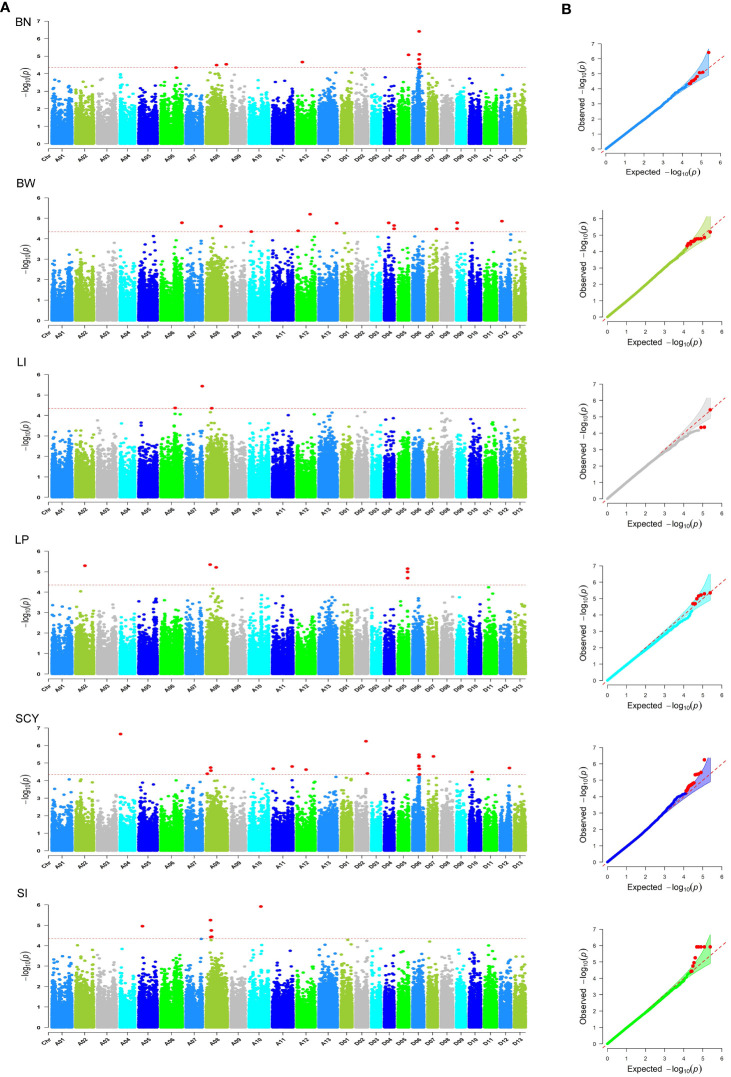
Summary of GWAS results for six yield-related traits (BN, BW, LI, LP, SCY, and SI) using BLUP data. **(A)** Manhattan plot for all six traits. The X-axis represents the chromosomal position of the SNPs in the cotton genome, and the Y-axis represents the −log10 of the P-values. The red dashed line indicates the significance threshold (−logP>4.5); **(B)** Q–Q plots for all six traits. The Y-axis represents the observed −log10 P-values, and the X-axis represents the expected -log10 P-values. The red points indicate significant SNP.

To identify the most suitable model for conducting GWAS analysis of our datasets (BLUPed traits and 2,41,086 SNPs), six statistical models (GLM, MLM, CLMM, MLMM, FarmCPU, and Blink) were compared using a quantile–quantile (Q–Q) plot (**Supplementary Figure S4**). From the Q–Q Plot, MLMM has the best fit followed by FarmCPU and Blink, whereas the GLM, CLMM, and MLM models deviated early from the expectation line. Based on the Q–Q plots, the MLMM model was selected as the best model for identifying significantly associated SNPs for the six studied yield-related traits. This underscores the importance of choosing an appropriate model for GWAS to avoid false positives and to increase the accuracy of the results. In total, 205 SNPs or quantitative trait nucleotides (QTNs) with 90, 67, and 48 in E1, E2, and BLUP, respectively were identified to be significantly associated with six traits above the significance threshold of −logP >4.5 (**Figure 5**, **Supplementary Figures S5, S6**; **Supplementary Table S6**). All SNPs were scattered unevenly, with 98 and 107 significant SNPs in the A and D sub-genomes, respectively. The maximum number of markers associated with the traits was found in Chr D06 (23), followed by Chr A08 (21), while Chr D13 did not contain any SNPs, and D03 had only one significant SNP. For BN, 28 significant SNPs were distributed on chromosomes A06, A08, A12, D01, D05, D06, D08, and D11, with the highest number in D11 (nine SNPs). Among these SNPs, NC_053442.1_45198190 had the highest positive phenotypic effect (3.107), with a −log_10_(P) value of 4.99, and NC_053441.1_63281905 had a negative effect (−3.606) with a −log_10_(P) value of 5.08. For BW, 27 significant SNPs were located on chromosomes A06, A08, A09, A10, A11 A12, A13, D04, D06, D07, D09, D10, D11, and D12, with the maximum number in A11 (five SNPs). Of these SNPs, NC_053432.1_54551322 showed a positive phenotypic effect (0.430) with a −log_10_(P) value of 5.49, and NC_053433.1_116352175 showed a negative effect (−0.465) with a −log_10_(P) value of 7.36. For LI, 50 significant SNPs were detected on chromosomes A01, A03, A04, A05, A06, A07, A11, A13, D01, D04, D06, D08, D09, D10, and D12 with six SNPs in A06. The phenotypic effect size of these SNPs ranged from 0.480 (NC_053429.1_20687114) to −0.47 (NC_053442.1_33992120) with −log_10_(P) values of 5.75 to 5.47, respectively. For LP, 40 significant markers were observed in 14 chromosomes of the cotton genome, with the maximum number in Chr D10 (nine SNPs). They explained the −2.072 (NC_053444.1_64810245) to 2.285 (NC_053441.1_58365634) range of phenotypic effect size having a P-value of 5.1 to 5.89. A total of 28 significant markers were observed for SCY located on 12 chromosomes, with the highest occurrence at D06 (nine SNPs), contributing 16.696 to −15.449 phenotypic effect size. Loci NC_053427.1_2009750 and NC_053431.1_72867934 had positive and negative effects on SCY, respectively, with −log_10_(P) values of 6.66 to 4.53, respectively. Similarly, SI had a total of 32 significant markers distributed on chromosomes A02, A05 (six SNPs), A07, A08, A10, A13, D01, D02, D07, D08, D09, and D12, explaining −0.876 to 0.959. The −log_10_(P) values of the loci contributing to positive effects (NC_053428.1_79218940) and adverse effects (NC_053431.1_70272704) were 5.45 to 4.99, respectively.

### 3.5 Identification and comparison of QTLs

According to the definition of QTLs from the previous methods (Sun Z. et al., 2017; Song et al., 2019), a total of 134 QTLs were obtained from 205 significant markers in this study, out of which maximum QTLs were identified for LI (30) followed by LP (25), SCY (21), SI (20), BW (18), and BN (12) traits (**Supplementary Table S7**). Similar to the significant markers, these candidate loci were also scattered among different chromosomes of the cotton genomes harboring 69 QTLs in the A subgenome and 65 in the D subgenome. Interestingly, most of the QTLs contained only one significant SNP, except for 37 QTL loci with more than one associated SNP. For instance, QTL (*qGhBN_D11-1*) had seven significant markers. All QTLs and GWAS signals for yield-related traits were retrieved from the database to compare our QTLs with those in previous reports. A total of 535 QTLs were reported for yield-related traits in 33 r QTL mapping studies. Similarly, details of the significant markers and their genomic positions were extracted from six reports of association mapping studies. Among the 134 QTLs identified in these studies, 39 were novel and newly identified in the Indian germplasm, and the remaining 95 QTLs overlapped with previously reported QTLs (**Supplementary Table S7**). In addition, we identified eight QTL that exhibited pleiotropic associations with more than one trait. Of these, three QTLs (*qGhBN_SCY_D6-1*, *qGhBN_SCY_D6-2*, *and qGhBN_SCY_D6-3*) on chromosome D06, were found to be associated with BN and SCY having common significant markers (NC_053442.1_36092628, NC_053442.1_37082185, NC_053442.1_37984052) within the same genomic intervals (35.8–38.1 Mb). Also, three QTLs (*qGhSI_LI_A5*, *qGhLI_SI_A13, qGhLI_SI_D9*) exhibited pleiotropic association for SI and LI, of these *qGhLI_SI_A13* had a common marker (NC_053436.1_78553469) for LI and SI while the other two had overlapping QTL intervals in terms of physical position (25.54–25.94 Mb in A05 and 5.67–6.07 Mb in D09). Moreover, BW and SCY had one pleiotropic QTL (*qGhBW_SCY_A10*) because of the presence of a common SNP marker (NC_053433.1_116352175) within genomic intervals of 116.15–116.55 Mb. In addition, one QTL (*qGhLP_BN_A8*) showed an association between LP and BN owing to the overlapping of QTL intervals in Chr A08 (59.74–60.14 Mb). As these traits showed a significant correlation at the phenotypic level (BN and SCY = 0.80, BW and SCY = 0.74, and SI and LI = 0.65), they were also found to have pleiotropic-associated markers at the genomic level. This result implies that a network of QTLs with multiple phenotypic effects may control fiber yield traits.

### 3.6 Identification of favorable haplotypes of pleiotropic QTLs

**Figure 6:**
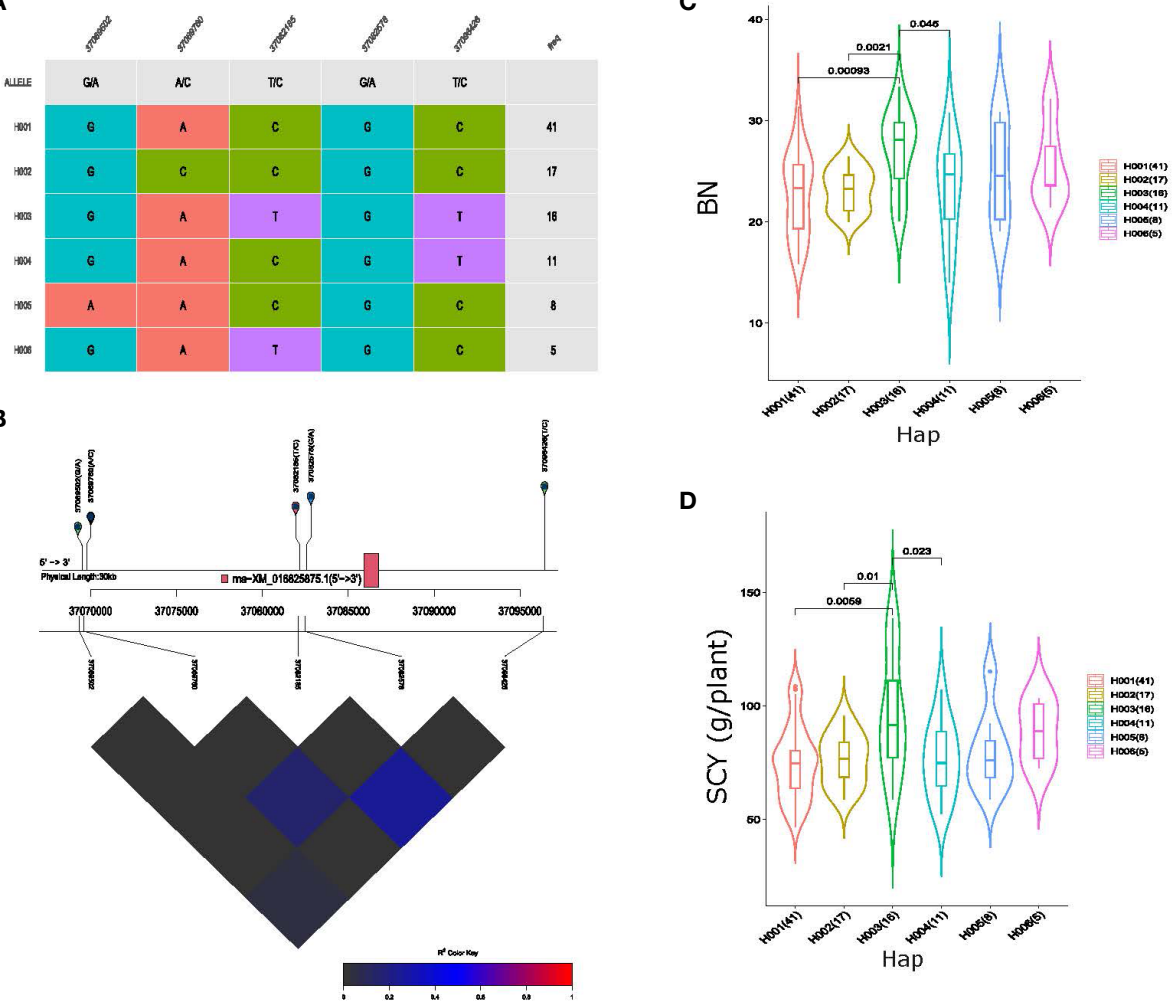
Haplotype analysis of pleiotropic QTL *qGhBN_SCY_D6-2*, **(A)** haplogroups observed in our 117-association panel using five SNP markers, **(B)** genomic location of five SNP loci and LD based on the pairwise R^2^ values between the SNPs estimated in Chromosome D06. The R^2^ values are indicated using the color bar. **(C, D)** Phenotypic differences of boll number (BN) and seed cotton yield (SCY) g/plant among the six haplogroups.

To identify the cumulative effects of favorable SNPs, haplotype analysis using eight QTL loci exhibiting pleiotropic associations was conducted. Haplotype analysis of QTL *qGhBN_SCY_D6-2*, associated with BN and SCY traits on Chromosome D06, formed a haplotype block with five SNP markers, which consisted of six haplogroups in our association panel (**Figure 6A**). All five markers showed substantial LD (**Figure 6B**), and variations in these haplotype alleles led to significant differences in the phenotypes of BN and SCY. The average BN values of the haplogroups were 22.72, 23.22, 27.05, 23.14, 24.92, and 25.63 per plant, respectively, and for SCY, the average values among the six haplogroups were 74.71, 76.25, 95.99, 76.34, 79.51, and 88.54 g/plant, respectively, for which H003 (haplotype 3) showed significantly higher BN and SCY values than those of the other haplogroups (**Figures 6C, D**). Haplotype analysis of *qGhSI_LI_A5* QTL resulted in the formation of four haplogroups with seven SNPs having substantial LD on Chromosome A05 (**Supplementary Figures S7A, B**) among the 117 cotton germplasms. The average LI and SI of H003 (haplotype 3) were 4.85 g and 8.56 g, respectively, higher than those of the other three haplogroups (**Supplementary Figures S7C, D**) showing significant phenotypic variation.

**Figure 7:**
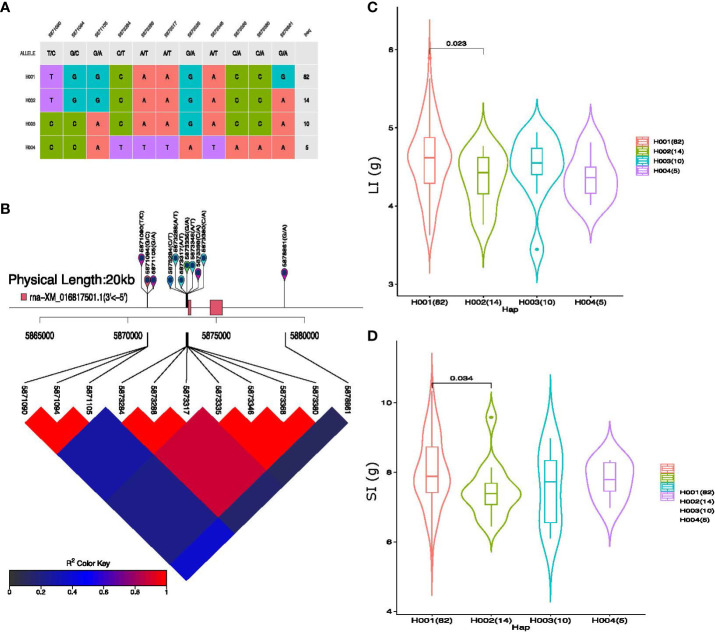
Haplotype analysis of pleiotropic QTL *qGhLI_SI_D9*. **(A)** Haplogroups observed in our 117-association panel using 11 SNP markers, **(B)** Genomic location of eleven SNP loci and LD based on the pairwise R^2^ values between the SNPs estimated in Chromosome D09. The R^2^ values are indicated using the color bar. **(C, D)** Phenotypic differences of lint index (LI) g and seed index (SI) g among the four haplogroups.

Eleven SNP markers were associated with pleiotropic QTL *qGhLI_SI_D9*, which represents four haplogroups in our cotton germplasm (**Figure 7A**). Substantial phenotypic variation for LI and SI and strong LD was observed among the 11 haplotype alleles present within these haplotype groups on Chromosome D09 (**Figures 7B–D**). The average LI values of the four haplotype groups were 4.59, 4.33, 4.48, and 4.36 (g), respectively, similarly, the average value of SI among the haplogroups was 8.00, 7.47, 7.57, and 7.76 (g), respectively. The average value of H001 (haplotype 1) was greater than that of the other haplotypes for both LI and SI. In addition, *qGhBW_SCY_A10* represented six haplogroups consisting of 22 SNP markers with strong LD (**Supplementary Figures S8A, B**). Among all the six haplogroups, haplotype 3 (H003) exhibits the highest average BW and SCY value of 3.56 g and 93.91 g/plant, respectively (**Supplementary Figures S8C, D**). Furthermore, the other four QTLs, *qGhBN_SCY_D6-1*, *qGhBN_SCY_D6-3, qGhLI_SI_A13*, and *qGhLP_BN_A8*, showed phenotypic differences; however, the haplotype alleles of these QTLs did not have any gene features within the 10–20 kb window.

### 3.7 Identification of candidate genes

**Figure 8:**
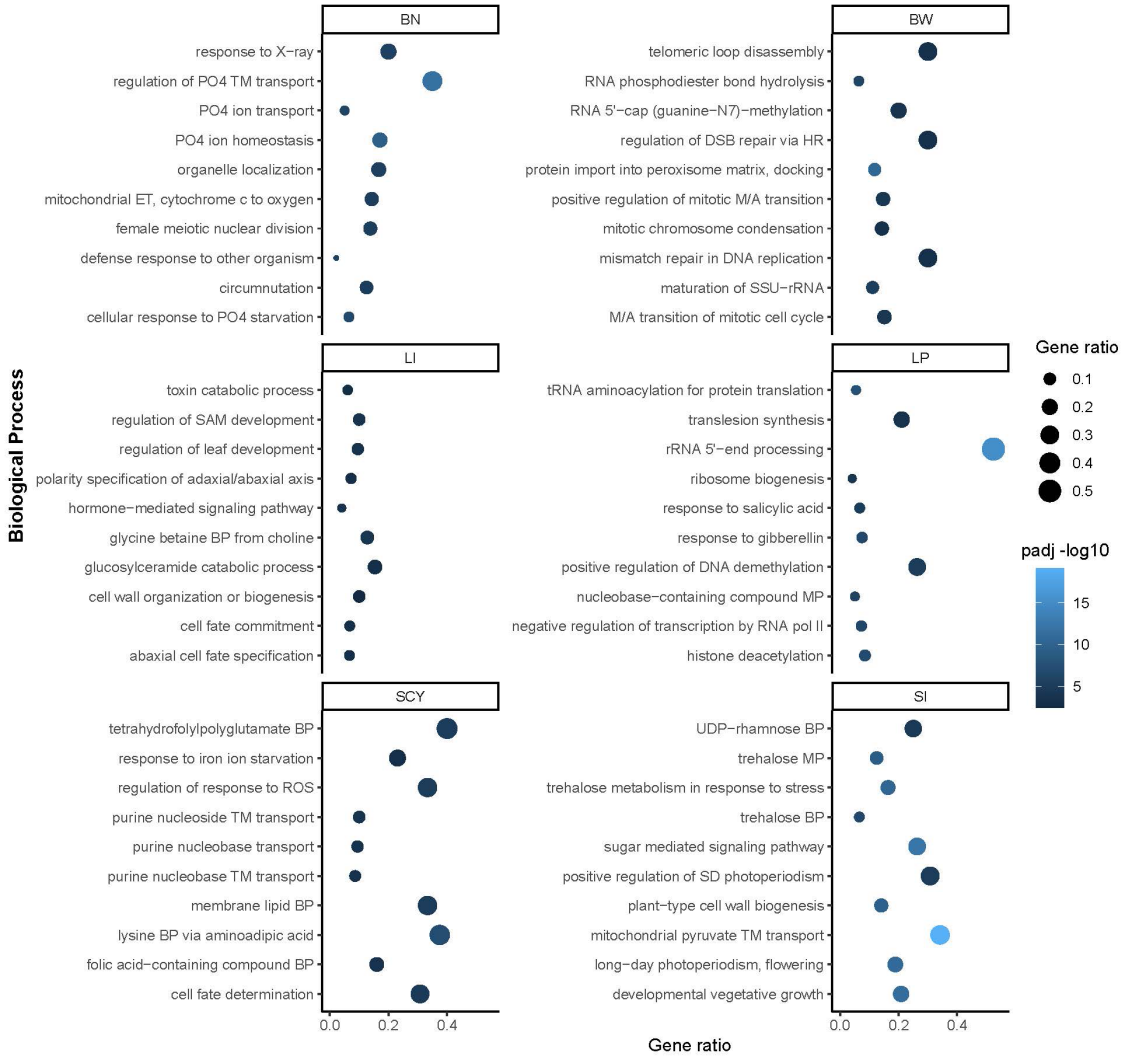
Biological process gene ontology term enrichment for genes located in the QTL region of yield-related traits. The top 10 BP ontology terms are shown for each trait. The y-axis represents the BP and the x-axis enrichment scores for each trait. The color scale shows the p-value of enrichment and bubble size enrichment score.

Genomic intervals of 134 reported QTLs were extracted and annotated to identify the putative candidate genes associated with each trait. Functional annotation of these regions retrieved 2,509 unique genes comprising 1,966 protein-coding, 277 lncRNAs, 204 snoRNAs, 30 tRNAs, 29 snRNAs, and three miscRNAs. The distribution of these genes in the QTL regions ranged from 1 to 65, except for *qGhLI_D4-1* and *qGhBN_SCY_D6-1*, which did not harbor any putative genes. Only 14 (~10%) QTLs had<5 putative genes, 33 QTLs (~24%) had >5–<10 putative genes, while all the remaining 85 QTLs (~63%) covered >10 genes (**Supplementary Table S7**). A total of 192, 432, 557, 591, 398, and 441 genes were associated with BN, BW, LI, LP, SCY, and SI, respectively. Moreover, the distribution of these genes across the cotton genome was uneven, with 1211 genes located in the A sub-genome and 1,298 genes in the D sub-genome, respectively. Chromosome D08 had the maximum number of genes (205 genes), whereas the minimum number of genes (13 genes) were in chromosome A13. Further gene ontology (GO) enrichment analysis for each trait was conducted to understand the function of each candidate gene, and it was found that these genes within the QTL regions were predominantly enriched for different biological processes. The top ten biological processes enriched for each yield-related trait are shown in **Figure 8**. For BN, the enrichment analysis results showed four terms that belong to phosphate metabolism: regulation, transport, homeostasis, and cellular response to phosphate starvation. BW includes terms related to RNA processing, DNA replication, maintenance, and mitosis, reflecting cell growth-associated processes enriched in this region. The LI region has genes associated with cellular fate determination, polarity specification, toxin catabolism, and cell wall organization. The LP region regulates gene expression at the epigenetic (DNA demethylation and histone deacetylation), transcriptional, and translation levels, and genes responsive to salicylic acid and gibberellin. SCY has stress-responsive, purine metabolism, and cell fate-related gene ontology. SI contains sugar signaling, trehalose synthesis and metabolism, plant cell wall synthesis, photoperiodism, and vegetative growth development-related genes enriched within its region.

**Figure 9:**
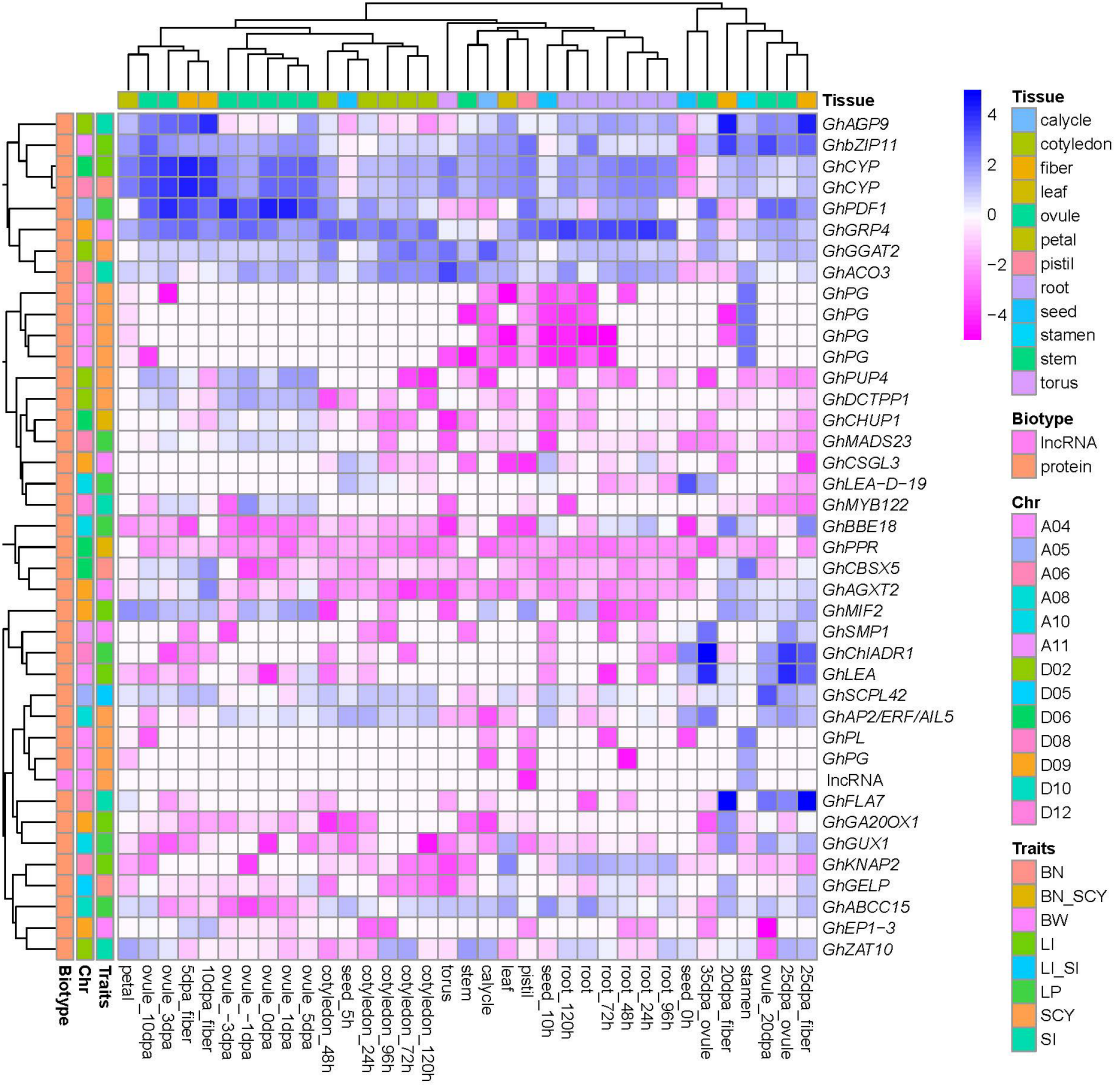
**A** heatmap of putative candidate genes for six yield traits preferentially active in the ovule, fiber, and seed tissues of cotton. The color scale in the heatmap represents the zFPKM transformed value. Column label is provided for type of tissue and row label is provided for gene biotype, chromosomal location of gene and candidate gene traits.

In addition, to identify the most active genes related to six yield traits, gene expression analysis was performed using RNA-seq data from diverse cotton tissues (seed, cotyledon, root, stem, leaf, torus, calycle, petal, stamen, pistil, ovule, and fiber). Of the 2,509 candidate genes, 870 were found to be transcriptionally active, showing a zFPKM value of ±3 in at least one tissue (**Supplementary Figure S9**). The numbers of active genes in different QTLs for BN, BW, LI, LP, SCY, and SI were 73, 169, 185, 208, 137, and 143, respectively. Furthermore, to identify the highly expressed active genes for yield-related traits, the expression profiles were mainly focused on the ovule, fiber, and seed tissues. For BN, *qGhBN_A6-1, qGhBN_D5-1*, *and qGhBN_D6-4* contained highly expressed active genes such as *GhCYP* (high expression at 5DPA of fiber development stage), *GhGELP* (highest expression at 20 DPA fiber development stage), and *GhCBSX5* (highly active in stamen tissue and at the 10 DPA of fiber development stage). For the BW trait, *qGhBW_D9-3* had two highly expressed genes (*GhAGX2* and *GhEP1-3*) showing higher expression at 10 and 20 DPA at the fiber development stage, respectively. In addition, the BW QTL *qGhBW_A11-1*, *qGhBW_D9-*1, and *qGhBW_D9-*2 contained the highly expressed gene *GhSMP1* (higher activity specifically at 25 and 35 DPA of ovule development), *GhGRP4* (expression in all stages of ovule development), and *GhCSGL3* (positive activity in seeds at 5 and 10 h), respectively. The *qGhLI_A4-2* QTL for LI has two genes, *GhbZIP11* (showing a higher expression profile in ovule and fiber development stages) and *GhLEA* (showing higher expression in ovule development). Other LI QTLS, *qGhLI_A6-1*, *qGhLI_D6-2*, *qGhLI_D9-1*, and *qGhLI_D9-2* show expression profiles for *GhKNAP2* (expressed in root tissue), *GhCYP* (showing higher activity in all tissues except seed), *GhMIF2* (higher activity in ovules and fibers at mid developmental stages), and *GhGA20OX1* (highest expression 20 DPA of fiber development) genes. Seven LP QTLs, *qGhLP_A5-1*, *qGhLP_A6-1*, *qGhLP_A10-1*, *qGhLP_A10-3*, *qGhLP_A10-6*, *qGhLP_D8-2*, and *qGhLP_D10-3* contained *GhPDF1* (highest activity in ovule and fiber developmental stages), *GhMADS23* (higher activity in ovule developmental stages), *GhGUX1* (expression at 20 DPA of fiber stage), *GhBBE18* (expression at 25 DPA of fiber stage), *GhLEA-D-19* (high expression at 0 and 5 h of seed development and 35 DPA of ovule development), *GhchlADH1*(showing higher expression in 20 and 25 DPA fibers, ovule 20 DPA, root, and leaf tissues), and *GhABCC* (higher activity in 20 and 25 DPA fiber, 20 DPA of ovule development along with root and leaf). The *qGhSCY_A4-1 QTL* of SCY contains seven genes (five *GhPG*, one *GhPL*, and one *GhlncRNA*) and showed interesting expression profiles that were highly expressed in stamen tissues in contrast to other tissues, especially the pistil. In addition, *qGhSCY_D2-1* for the SCY trait had two expressed genes (*GhPUP4* and *GhDCTPP1*) that were highly expressed in ovules at all stages (except at 35 DPA). The other two SCY QTLs (*qGhSCY_A8-2* and *qGhSCY_D2-2*) showed a high expression profile for *GhAP2/ERF_AIL5* (highly expressed in the later stage of ovule and fiber development along with all stages of seed development) and *GhGGAT2* (expressed 25 and 35 DPA of fiber development), respectively. For *SI*, *qGhSI_D2-3* contains two genes, *GhAGP9* (highest activity at all stages of fiber development) and *GhZAT10* (activity at 25 DPA of both ovule and fiber development). Similarly, the *qGhSI_D8-2 QTL* for SI had two active genes, *GhACO3* and *GhFLA7* (which showed the highest activity during the ovule and fiber development stages). In addition, *qGhSI_D12-1* contained *GhMYB22*, which showed higher expression at an early stage of ovule development (−1 to 1 DPA). In addition, of the eight pleiotropic QTLs, two QTL *qGhBN_SCY_D6-3* and *qGhSI_LI_A5*, governed three active genes. *qGhBN_SCY_D6-3* had two active genes, *GhPPR* (higher expression in ovules at −3, 0, 3, and 5 DPI), and *GhCHUP1* (highest activity at 5 and 10 DPA of fiber development and 20 and 25 DPA of ovule development) while *qGhSI_LI_A5* had *GhSCPL42* (higher expression at 5, 10, and 20 DPA of ovule development). Of the 870 active genes, 40 candidate genes were selected for six yield-related traits that showed more contrasting expression profiles in the ovule, fiber, and seed developmental stages in comparison to other cotton tissues (**Figure 9**). Additionally, among the 40 genes significantly expressed in cotton yield traits, 15 genes were reported to be associated with different fiber development stages, eight genes were reported for different biotic and abiotic stresses, and 17 genes were novel genes associated with fiber development (**Supplementary Table S8**).

## 4 Discussion

Cotton is one of the most important natural fibers and is a raw material for many textile industries. Its yield can significantly influence the textile industry because it is directly related to overall supply and demand dynamics, production capacity, pricing, innovation, global trade, and sustainability efforts within the industry. Cotton yield is a complex quantitative trait governed by multiple yield-related descriptors that are difficult to improve simultaneously using traditional breeding methods (Wang et al., 2019). Thus, the identification and characterization of genetic factors for targeted traits and their manipulation through conventional breeding coupled with genomic tools have consistently been the primary goals for cotton breeders, enabling a more precise selection of genotypes in the pursuit of developing varieties with higher yield potential. In the present study, GWAS was performed based on the natural population of 117 Indian upland cotton germplasms and genotyping-by-sequencing methods to deploy markers for six yield-related traits. Association mapping study is an effective tool for mapping complex quantitative traits to identify of key genes associated with such traits in many plants (Huang et al., 2012; Horton et al., 2014; Crowell et al., 2016; Varshney et al., 2019b; Wang et al., 2020). It is an analytical method and its ability to resolve associated loci relies on factors such as the size of the experimental population, marker density, and the selection of appropriate statistical tools or GWAS models for marker-trait identification (Liu et al., 2016). Population size has a great impact on association mapping studies, and it has been reported that a relatively large population size ensures sufficient genetic variation, thereby influencing the detection of significant QTLs (Su et al., 2016; Huang et al., 2017). Population sizes ranging from 95 to 800 have been previously studied in upland cotton in association studies (Gapare et al., 2017; Sun Z. et al., 2017; Dong et al., 2019; Kumar et al., 2021). Although our population size of 117 released varieties from different agro-climatic zones was not sufficiently large, they are suitable for mapping analysis because they stabilize after many years of genetic recombination (Nordborg et al., 2002). Along with population size, phenotypic variances in a single environment affect the accuracy of phenotypic data, thereby affecting the reliability of the mapping study. To rectify this error, the use of multi-environment and unbiased prediction data in association analysis has been proven effective in previous studies (Roorkiwal et al., 2018; Tomar et al., 2021). Phenotyping from multiple field locations effectively eliminates the influence of the environment and aids in the interpretation of environment-specific as well as general QTL (Gutiérrez et al., 2015). In the present study, we used two sites for the evaluation of six yield-related phenotypic traits: Punjab Agricultural University, Punjab (E1), and Tamil Nadu Agricultural University (E2), which are the two main cotton-growing belts in India. In addition, there is a large difference in the geographical position as well as the climatic conditions between these two zones (https://www.cicr.org.in/pdf/long_staple), and all six traits exhibited abundant phenotypic variation ranging from 6.32% to 24.7% and 6.17% to 20.1% in E1 and E2, respectively. Our findings also showed the presence of medium to high heritability (>50%, except for LP in E2), illustrating the importance of these germplasm lines for genetic improvement and showing the same phenotypic expression if sown in the same environment (Bhat et al., 2022). High and stable heritability has always been proven to be useful in determining the strong association between markers and traits of interest (Courtois et al., 2013). In addition, the correlation study among the traits showed a significant positive correlation between SCY and BN, and BW, and a maximum negative correlation between LP and SI (**Figure 2**) which corroborates the findings of Dong et al. (2018) and Sun et al. (2018). Thus, the presence of significant variation among the genotypes in different environments along with the strong correlation led us to explore markers associated with six yield-related traits through GWAS analysis.

Association mapping using various SSR or SNP chips has been previously reported in upland cotton (Mei et al., 2013; Gapare et al., 2017; Handi et al., 2017; Huang et al., 2017; Kumar et al., 2021); however, the development of high-resolution sequencing technologies such as GBS, SLAF-seq, and RAD-seq offers significant advantages and has led to tremendous progress in the development of numerous SNP markers for genetic mapping in cotton (Islam et al., 2016; Su et al., 2016; Geng et al., 2020; Wang et al., 2021) and other crops (Arruda et al., 2016; Maldonado et al., 2019; Ravelombola et al., 2021). Genotyping by sequencing (GBS) provides a comprehensive view of the genome by generating genetic markers that spread across the entire genome. In contrast, SSR markers and SNP chips have limited coverage, and may not capture the full genetic variation present in a population. This increased marker density improves the resolution and efficiency of GWAS, enabling the detection of more genetic associations and fine mapping of genomic regions. In addition, SNP chips often rely on preselected SNPs that may not fully represent the genetic diversity of a particular population or species, conversely, GBS allows for unbiased genotyping, reduces ascertainment bias, and enables a more accurate assessment of genetic variation. GBS technologies have enabled the rapid sequencing and genotyping of breeding populations, allowing plant breeders to accomplish genomic diversity, GWAS, genomic selection (GS), and marker development in many species without prior knowledge of the species genomes (Poland and Rife, 2012). In our study, a substantial number of high-quality SNP markers (2,41,086) were identified using the GBS method with an average density of 1SNPs/11.36 kb. The number of markers reported in our study was relatively higher than that reported by Wang et al. (2021); however, it was almost consistent with the findings of Geng et al. (2020). The higher number of markers in the current study was sufficient to conduct GWAS analysis, offering potential advantages in uncovering additional prominent loci and candidate genes (Wang et al., 2018).

Upland cotton has an extensive and intricate history of domestication and breeding, with a narrow genetic background, and is mostly influenced by geographical isolation and gene flow (Huang et al., 2017). Furthermore, understanding the population structure and relatedness in the association mapping panel is crucial for elucidating the heterogeneity of the genetic architecture and controlling false associations (Lu et al., 2015). Therefore, it is crucial to consider the population structure and degree of relatedness among individuals in association-mapping studies. In our study, the association panel was categorized into two clades (CGP1 and CGP2) based on molecular analyses using STRUCTURE software but were not completely consistent according to the geographical origin, as reported in previous studies (Gapare et al., 2017; Sun Z. et al., 2017; Song et al., 2019). In addition, clustering analysis using Nei’s genetic distance, kinship matrix, and PCA calculation methods showed good consistency with population structure analysis. The absence of geographical correlation in our germplasm might be attributed to the relatively high level of gene exchange and interspersed introduction or crossbreeding among the germplasms across different geographical regions of India. Overall, these results highlighted that the germplasms were not highly structured and exhibited weak relatedness, but considering the greater continuous phenotypic variations among the six yield-related traits (**Table 1**), the association population was further used in the GWAS analysis (Yano et al., 2016; Sun Z. et al., 2017; Song et al., 2019).

Several studies have indicated that effective control of the discovery of false positives resulting from population structure in crops may not be entirely achieved (Hamblin et al., 2011; Lipka et al., 2015). Therefore, to address these errors, we investigated various statistically robust models for the genome-wide association study using the GAPIT package in the ‘R’ programming. The strategy of utilizing two or more different models to identify significant markers in cotton has been reported in several studies (Badigannavar and Myers, 2015; Abdullaev et al., 2017; Sethi et al., 2017; Li C. et al., 2018; Su et al., 2018). In the present study, we implemented six different models; three univariate models (GLM, MLM, and CLMM) and three multivariate mixed models (MLMM, FarmCPU, and Blink). According to Segura et al. (2012), the use of a multivariate GWAS method addresses the issue of confounding between covariates and the test marker, and lowers the false discovery rate (FDR) compared to univariate GWAS when employing the same threshold, enabling the detection of a greater number of QTLs. By examining the Q–Q plots, we determined the most suitable models, and our findings demonstrated that the MLMM exhibited the best-fit model for our trait data, unlike the other models that exhibited early deviations from the expected line. The MLMM model has also shown successful results in other plants, such as Cannabis (Watts et al., 2021), tomato (Zhao et al., 2022), apricot (Omrani et al., 2019), and wheat (Mihalyov et al., 2017). Detected by the MLMM, 205 significant SNP from two environments and BLUP were found to be associated with six yield-related traits (**Supplementary Table S6**). These markers were unevenly distributed on all chromosomes of cotton (except for Chr D13), suggesting that the regulation of these traits involves a complex gene network. This finding aligns with those of previous reports, highlighting the intricate genetic control of cotton yields (Rong et al., 2007; Sun et al., 2018). Using these significantly associated markers, QTL were determined within the ±200 kb upstream and downstream regions, as reported previously (Sun Z. et al., 2017; Song et al., 2019). In this study, 134 QTLs were derived from 205 significant markers (**Supplementary Table S7**). Interestingly, some of these significant SNPs or QTLs were associated with multiple yield-related traits at pleiotropic loci. It has been reported that complexity and linkage between complex traits are common in many crops (Van Tienderen et al., 1996; Yan et al., 2011; Raman et al., 2019; Li A. et al., 2022). In upland cotton, four pleiotropic loci related to fiber yield, fiber quality, and flowering date have been reported by Wang et al. (2021). Similarly, four and nine pleiotropic loci have been reported for concurrent alterations in both lint yield and fiber quality traits in cotton (Geng et al., 2020; Li Y. et al., 2023). In our study, eight QTLs (six within the same genomic interval and two overlapping with adjacent QTLs) were found to be associated with six yield-related traits. QTL *qGhBN_SCY_D6-1*, *qGhBN_SCY_D6-2*, *qGhBN_SCY_D6-3* was simultaneously associated with BN and SCY, having common significant markers NC_053442.1_36092628, NC_053442.1_37082185, NC_053442.1_37984052. Three other QTLs, *qGhSI_LI_A5*, *qGhLI_SI_A13*, *and qGhLI_SI_D9*, were concurrently associated with SI and LI, of which *qGhLI_SI_A13* had a common marker (NC_053436.1_78553469), while the other two were overlapping QTLs. Similarly, *qGhBW_SCY_A10 and qGhLP_BN_A8* were pleiotropic QTLs for BW/SCY and LP/BN, respectively. Additionally, correlation analysis of these yield-related traits showed a significant positive correlation (between SCY and BW, BN), and the maximum negative correlation between LP and SI indicated that these are the favorable loci that could be the genetic foundation for the correlation between these traits. Thus, the pleiotropic loci identified in this study provide information to explore the molecular mechanism that explains the simultaneous enhancement of six intricate yield-related traits in cotton, which will be helpful in the selection of germplasms in crop improvement programs for yield traits in Indian upland cotton.

In the past few decades, hundreds of QTLs or GWAS signals associated with cotton fiber yield have been identified in different intraspecific and interspecific populations through various linkage and association mapping methods (Said et al., 2015; Huang et al., 2017; Ma Z. et al., 2018). Of the 134 QTLs detected in the present study, 95 QTLs overlapped within the genomic interval or were adjacent to the genomic position of the QTLs and GWAS signals identified in a previous study (**Supplementary Table S7**). Interestingly, some of the QTLs were trait-specific, as reported earlier within the same genomic interval. For instance, QTLs (*qGhBN_D5-1*, *qGhBN_D6-1*, *qGhBN_D6-2*, *qGhBN_D6-3*, and *qGhBN_D6-4*) for BN overlap within *TM58714_TM58742_TM58749* (Zhu et al., 2021), and *qBPP-1-43.5* (Zhang et al., 2011). Similarly, for BW, QTLs (*qGhBW_A13-2*, *qGhBW_D4-2*, *qGhBW_D7-1*, *qGhBW_D9-3*, *qGhBW_D12-1*) showed consistent results with *TM47610_TM47614*, *TM56685_TM56686, TM63749* (Zhu et al., 2021), *qBW-C16-1* (Wu et al., 2009) i15830Gh (Sun et al., 2018), and *qBW-06A-c26-1* (Yu et al., 2013b) BW QTLs reported previously. LI (*qGhLI_D6-2*, *qGhLI_D9-2*) corresponds to *QTLs qLI-D6-1* (Liu et al., 2012) and Br7_Lt%_23 (3,47+) (Lacape et al., 2013) LI QTLs reported in previous studies. For LP, three QTL (*qGhLP_A6-2*, *qGhLP_D6-1*, and *qGhLP_D8-1*) matched with the LP GWAS signals reported by Ma Z. et al. (2018) (*A06_102555770*), and QTL reported by Yu et al. (2013a) (*F2:3-qLP-c25-1*), and Chen et al. (2010) (*qLP-D8-1/qLP-F2:3-JES-1a*), respectively. Four QTLs (*qGhSCY_A13-*1, qGhSCY*_D6-*3, qGhSCY*_D2-1*, and *qGhSCY_D2-2*) were associated with SCY in the present and in the previously reported SCY QTLs: *qSCY-07A-c13-1*, *qSCY-06A-c25-1* (Yu et al., 2013b), *qSY-D2-1* (Wang et al., 2007), and *TC-qSCY-c14-1* (Yu et al., 2013a), respectively. Interestingly, pleiotropic QTLs for BN and SCY (*qGhBN_SCY_D6-1*, *qGhBN_SCY_D6-2*, and *qGhBN_SCY_D6-3*) showed congruency for both BN (*qBPP-1-43.5*) and SCY (*qSCY-06A-c25-1*) traits, as reported previously (Zhang et al., 2011; Yu et al., 2013b). Pleiotropic QTL (*qGhBW_SCY_A10* and *qGhLI_SI_D9*) were consistent with the BW association signal *A10_99131954* (Ma Z. et al., 2018) and *qSI-D9-1* (Shen et al., 2007) located on chromosomes A10 and D09, respectively. Therefore, our findings corroborate those of previous studies, validate the authenticity of current GWAS results, and increase confidence in the reliability of some QTLs/SNPs. These QTL/SNPs, exhibit stable inheritance and were consistently detected across diverse segregating populations with varying genetic backgrounds and through different mapping methods, and have a significant potential for future breeding programs aimed at improving cotton yield in India.

High-quality allelic loci are precious assets for agricultural breeding initiatives, and the identification of favorable alleles is an efficient approach to enhance the traits within crop plants (Su et al., 2016). Many recent studies have identified haplotype alleles for important traits, such as haplotype analysis of yield-related traits in soybean (Bhat et al., 2022), agronomically important traits in Arabidopsis (Lu et al., 2019), grain quality traits in rice (Wang et al., 2017), high thousand-kernel weight in wheat (Sun C. et al., 2017), grain yield, and flowering time under drought and heat stress conditions in maize (Yuan et al., 2019), which have shown considerable potential for the identification of traits and crop improvement. In cotton, Song et al. (2019) reported the haplotype analysis of two significantly associated SNPs with the lint percentage (LP) trait had a positive effect on LP, and these favorable alleles can be pyramided in a target line by marker-assisted selection. Similarly, two major haplotypes for fiber length and strength in cotton have been identified on chromosomes Dt11 and At07 (Sun Z. et al., 2017). In the present study, haplotype analysis of pleiotropic QTLs identified for different traits was conducted using a 10–20 kb window. The combination of favorable alleles identified within H003 (haplotype 3) was significantly higher for BN, BW, SCY, LI, and SI in the pleiotropic QTL *qGhBN_SCY_D6-*2, qGhSI*_LI_A5*, and *qGhBW_SCY_A10*, respectively. Eleven SNP combinations within haplogroup H001 were greater than other haplotype groups in the pleiotropic QTL *qGhLI_SI_D9*. Our results revealed that the combination of favorable alleles led to the identification of haplogroups that regulate a diverse range of phenotypes and significant phenotypic variation in yield-related traits in cotton. All identified diverse haplotypes can be further pyramided as a targeted line by marker-assisted breeding in cotton breeding programs. Thus, haplotype-based breeding strategies will aid in choosing favorable plant genotypes that carry advantageous haplotype alleles that have great potential for crop improvement (Varshney et al., 2019a).

Several genes associated with yield traits, such as *Gh_D08G2376* (Huang et al., 2017), *Gh_D12G2344* (Sun et al., 2018), *Gh_D05G1124* (Song et al., 2019), *Gh_A02G1268* (Su et al., 2016), *Gh_D02G0025* (Ma Z. et al., 2018), *AHP5* (Fang et al., 2017), *Gh_A02G0111* (Niu et al., 2023), and *Ghir_A08G009110* (Feng et al., 2022) have been previously reported in GWAS using different association mapping studies. In the present study, 2,509 candidate genes were identified within the confidence interval of the identified QTLs. Previous studies have suggested that genes preferentially expressed at different stages of fiber, ovule, and seed development may be involved in fiber yield and quality (Huang et al., 2017); therefore, we selected 40 highly active genes showing significant expression profiles (**Supplementary Table S8**). For BN, three candidate genes were identified; the *GhGELP* isoform has been reported to have a direct function in ovule, fiber, and seed development in cotton (Ma R. et al., 2018). *GhCBSX5* has reported to be involved in other biological processes (Ali et al., 2021); however, its role in fiber development in cotton has not yet been reported. *GhCYP* has no functional information in cotton; its isoform has been reported to play a significant role in drought tolerance in wheat (Zang et al., 2010). Gene annotation of the BW reported QTL intervals identified five active genes. *GhCSGL3* plays a direct role in secondary cell wall biosynthesis leading to enhanced lint yield and quality in cotton (Li et al., 2015; Zhang et al., 2015), whereas *GhAGXT2* and *GhEP1-3* play a positive role in abiotic stress in cotton (Li L. et al., 2022). Two other genes, *GhSMP1* and *GhGRP4*, were reported to be novel genes governing boll weight traits in the present study. Six candidate genes have been reported for LI traits of which two genes (*GhKNAP2* and *GhGA20OX1*) were functionally validated to play a major role in the cotton fiber development process (Xiao et al., 2010; Gong et al., 2014). The other two genes (*GhLEA* and *GhbZIP11*) have been reported to be stress-responsive in cotton (Liang et al., 2016; Magwanga et al., 2018). In addition, the functional roles of *GhCYP and GhMIF2* have not been reported in cotton, but its isoform has been reported to play a vital role in petal elongation in *Gerbera hybrida* (Han et al., 2017). Similarly, expressional analysis of candidate genes for LP traits resulted in the identification of seven active genes. Three genes (*GhLEA-D-*19, *GhPDF1*, and *GhABCC15*) have direct roles in cotton seed germination, fiber cell initiation, and elongation (Dure III and Galau, 1981; Zhu et al., 2003; Deng et al., 2012). The other four genes (*GhGUX1*, *GhBBE18*, *GhMADS23*, and *GhChlADR1*) were newly identified genes and might play a vital role during fiber development based on their expression profile; however, the functions of these in cotton remain to be further explored. Interestingly, for SCY highest number of genes was found (eleven) out of five *GhPG*, one *GhPL*, and one lncRNA reported encodes the highly significant marker (NC_053427.1_2009750) showing the highest phenotypic effects (~16.69%) could be the most promising genes found in the present study that could play a major role in fiber yield trait, which also corroborates with previously reported studies (Li Z. et al., 2023; Sun et al., 2020). Another gene, *GhAP2/ERF/AIL5*, has also been reported to play a key role in the growth and development of cotton plants (Zafar et al., 2022). *GhPUP4*, *GhDCTPP1*, and *GhGGAT2* have no direct role in cotton; however, *GhPUP4* has been reported to play a major role in the enhancement of grain size increase in rice (Xiao et al., 2019). SI has five active genes, (*GhMYB22*, *GhAGP9*, *GhZAT10*, *GhACO3*, and *GhFLA7*), which play an active role in fiber initiation elongation and freezing tolerance, as reported previously (Shi et al., 2006; Huang et al., 2013; Li P. et al., 2023). Additionally, pleiotropic QTLs (*qGhBN_SCY_D6-*3 and *qGhSI_LI_A5*) have three active genes (*GhPPR*, *GhCHUP1*, and *GhSCPL42*), whose functions in cotton fiber development have not been deciphered; however, their isoforms have been reported to play a functional role in defense mechanisms against abiotic and biotic stress (Wang et al., 2022). All 23 stable and 17 novel genes identified in the current study exhibited a high expression profile, which renders them promising candidate genes for future investigations and their functional validation would reveal their role in cotton yield improvement through functional genomics approaches. In conclusion, the present study unveiled a rich source of genetic elements, including SNPs, QTLs, and putative candidate genes associated with fiber yield traits in Indian upland cotton.

## 5 Conclusion

The present study has made significant strides in understanding the genetic structure and diversity of the Indian cotton germplasm, the identification of SNPs associated with fiber yield traits, and the subsequent identification of potential candidate genes. A weak population structure in the Indian cotton germplasm revealed two subgroups when the population structure was analyzed using a variety of approaches, including PCA, NJ tree, and kinship analysis. A low level of genetic relatedness among the genotypes was observed in the kinship matrix, which is required for breeding programs because it preserves the genetic diversity with wide adaptability. A total of 205 significant SNPs associated with six yield-related traits were identified through the GWAS and further delineated into 134 QTLs. Interestingly, several of these QTLs showed pleiotropic effects, indicating that these loci govern several traits and are thus advantageous candidate loci for introduction into breeding programs aimed at enhancing fiber yield. Additionally, 2,509 unique candidate genes were identified within the vicinity of these QTLs. The biological processes of the trait-associated genes were revealed using gene ontology enrichment analysis. While analyzing the public domain RNA-seq data, we identified 40 potential candidate genes across various cotton fiber developmental stages, several of which are known to be associated with fiber yield, while others need further functional validation to decipher their role in cotton yield. In conclusion, the present study unveiled a rich source of genetic elements, including SNPs, QTLs, and putative candidate genes associated with fiber yield traits in Indian upland cotton. These findings provide a solid foundation for further research on the functional roles of these genetic elements and their potential utilization in breeding programs to improve cotton fiber yield in India. To clearly determine the involvement of candidate genes governing cotton yield traits, further functional validation is necessary. It is anticipated that this effort will make a substantial contribution to the MAS breeding of high-fiber-yielding cotton varieties, thereby enhancing cotton farming productivity and the sustainability of the Indian cotton industry.

## Data availability statement

The datasets presented in this study can be found in online repositories. The names of the repository/repositories and accession number(s) can be found below: NCBI SRA accession number: PRJNA982386.

## Ethics statement

The manuscript number allotted by the ethical committee is CSIR-NBRI_MS/2023/06/10.

## Author contributions

SNJ and SVS conceptualized the idea of the project. NMB and HK performed field cultivation of cotton germplasms at TNAU, Coimbatore, Tamil Nadu, and PAU Faridkot, Punjab, respectively. BJ conducted the phenotyping and performed all the experiments, whereas GJT participated in phenotyping only. SS, BJ, SJ, and MI conducted all the computational and statistical data analyses. BJ wrote the first draft of the manuscript and SS helped into it. GJT, NMB, DA, SJ, DK, SVS, and SNJ revised the manuscript and provided various suggestions. BJ includes all the corrections and suggestions in the final manuscript. All authors contributed to the article and approved the submitted version.
