# Supplementary material for: Genome-wide association study of fiber yield-related traits uncovers the novel genomic regions and candidate genes in Indian upland cotton (Gossypium hirsutum L.)
Source: Front Plant Sci. 2023 Oct 23;14:1252746. doi: 10.3389/fpls.2023.1252746 (PMC10630025; doi:10.3389/fpls.2023.1252746)
Supplement: Supplementary file 1 [file DataSheet_1.zip › Supplementary Data/Supplementary figures.docx]

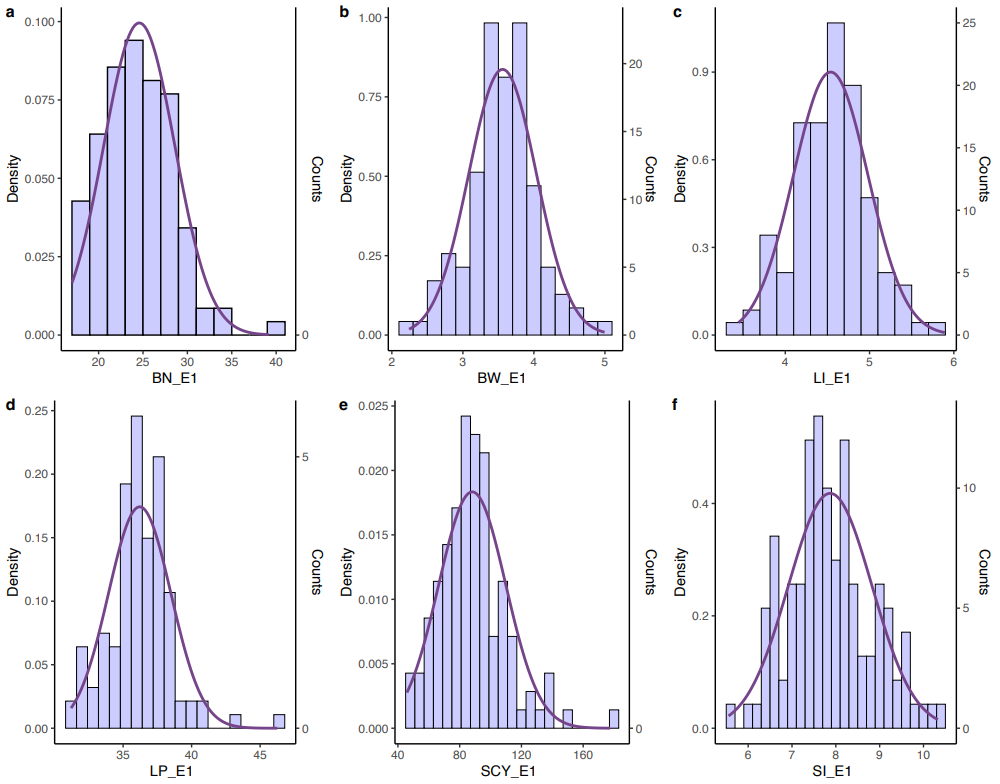


**Figure S1.** Frequency distribution of six yield-related traits in E1 environment; (a) Boll number (BN); (b) Boll Weight (BW); (c) Lint Index (LI); (d) Lint Percentage (LP); (e) Seed Cotton Yield (SCY); (f) Seed Index (SI)


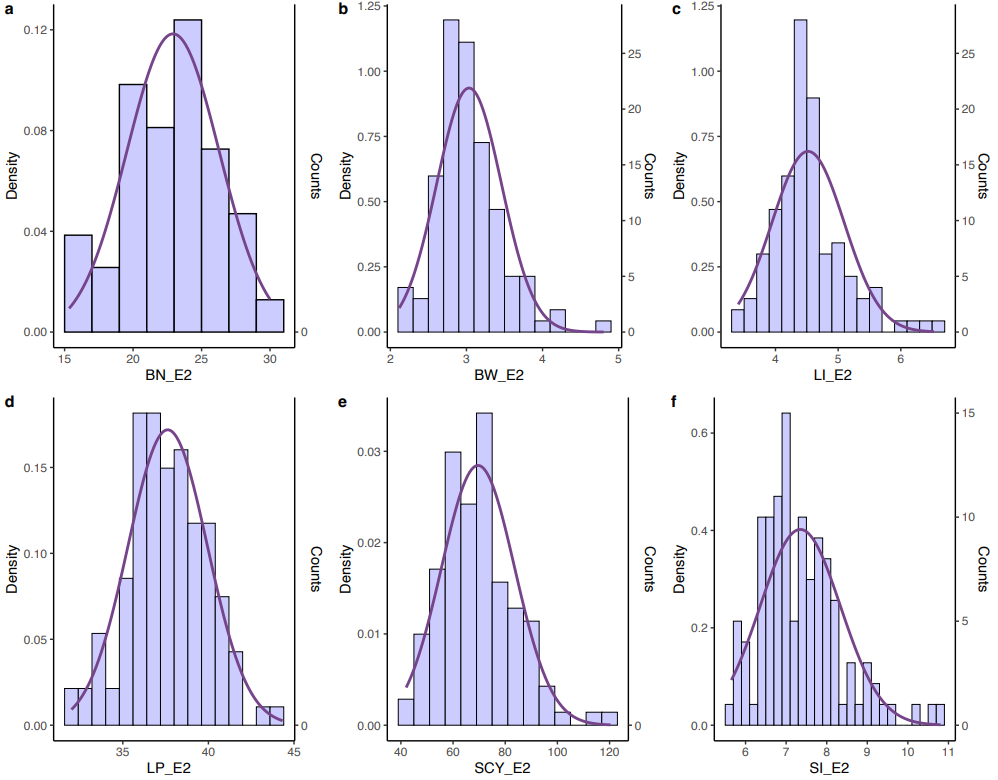


**Figure S2.** Frequency distribution of six yield-related traits in E2 environment; (a) Boll number (BN); (b) Boll Weight (BW); (c) Lint Index (LI); (d) Lint Percentage (LP); (e) Seed Cotton Yield (SCY); (f) Seed Index (SI)

**Figure S3.** Frequency of pair-wise relative kinship coefficients


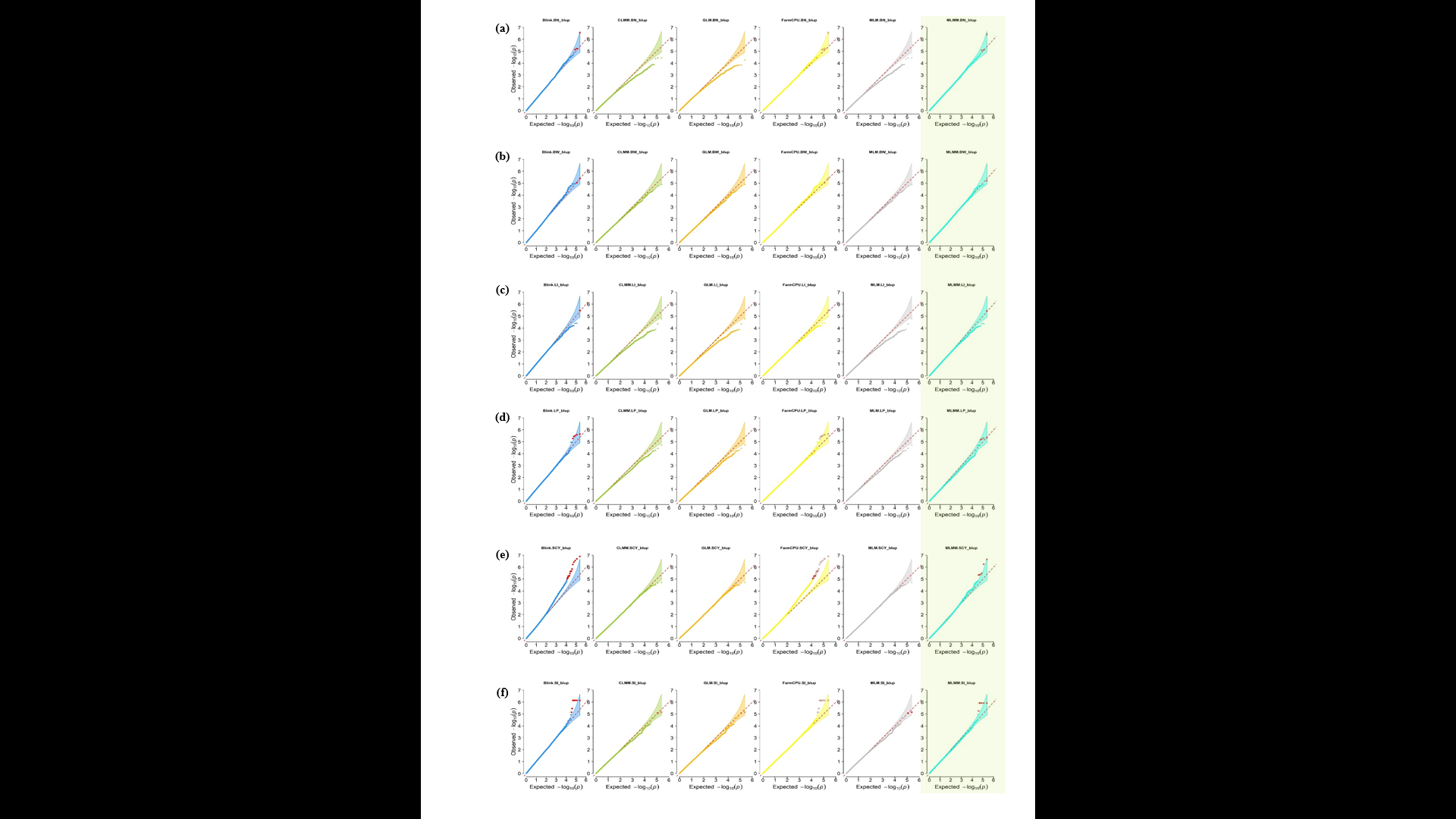


**Figure S4.** The Q-Q plot of BLUP estimated value with six methods (GLM, MLM, CLMM, MLMM, FarmCPU and Blink) using GAPIT. Highlighted QQ plot is the best-fitted model (MLMM); (a) Boll number (BN); (b) Boll Weight (BW); (c) Lint Index (LI); (d) Lint Percentage (LP); (e) Seed Cotton Yield (SCY); (f) Seed Index (SI)


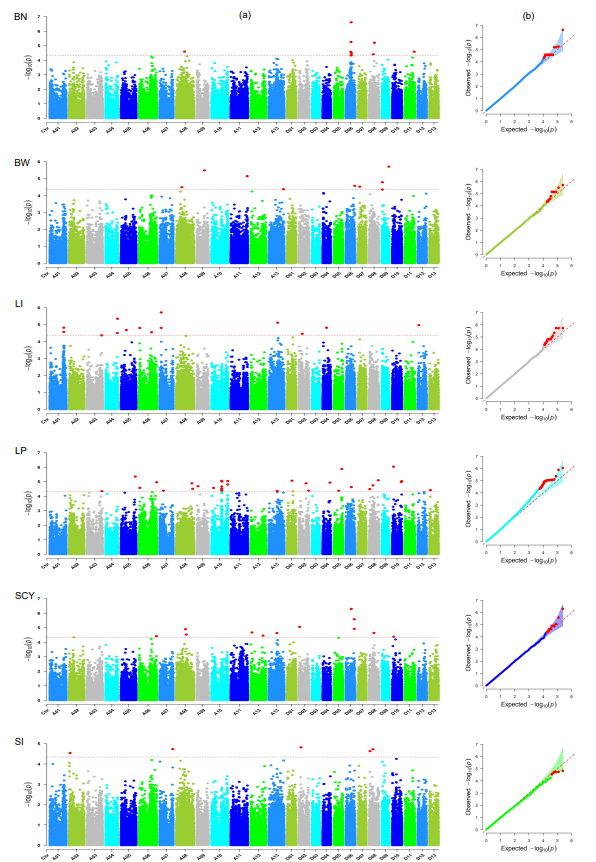


**Figure S5.** Summary of GWAS results for six yield-related traits (BN, BW, LI, LP, SCY, and SI) at E1. (a) Manhattan plot for all the six traits. The red dashed line indicated the significance threshold (-logP>4.5); (b) Q-Q plots for all six traits using the MMLM model in GAPIT


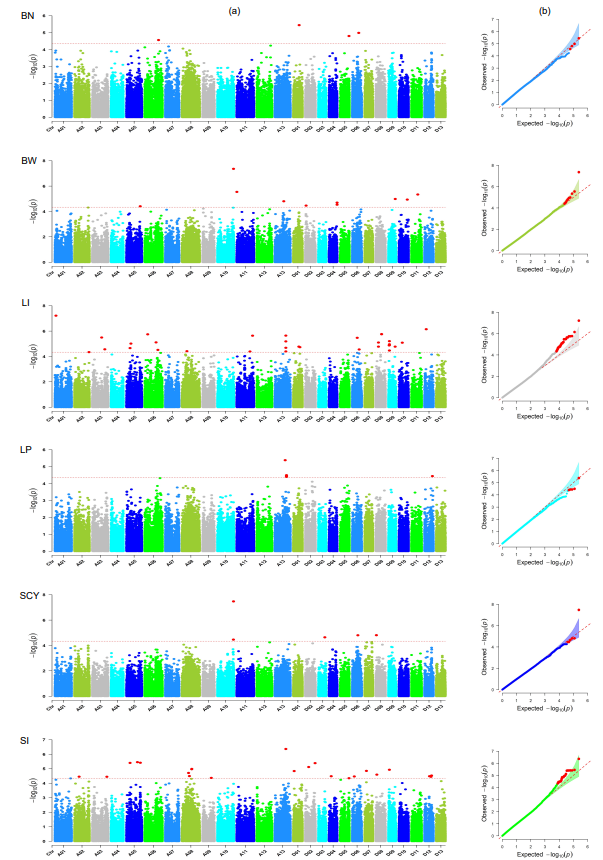
**Figure S6.** Summary of GWAS results for six yield-related traits (BN, BW, LI, LP, SCY, and SI) at E2. (a) Manhattan plot for all the six traits. The red dashed line indicated the significance threshold (-logP>4.5); (b) Q-Q plots for all six traits using the MMLM model in GAPIT


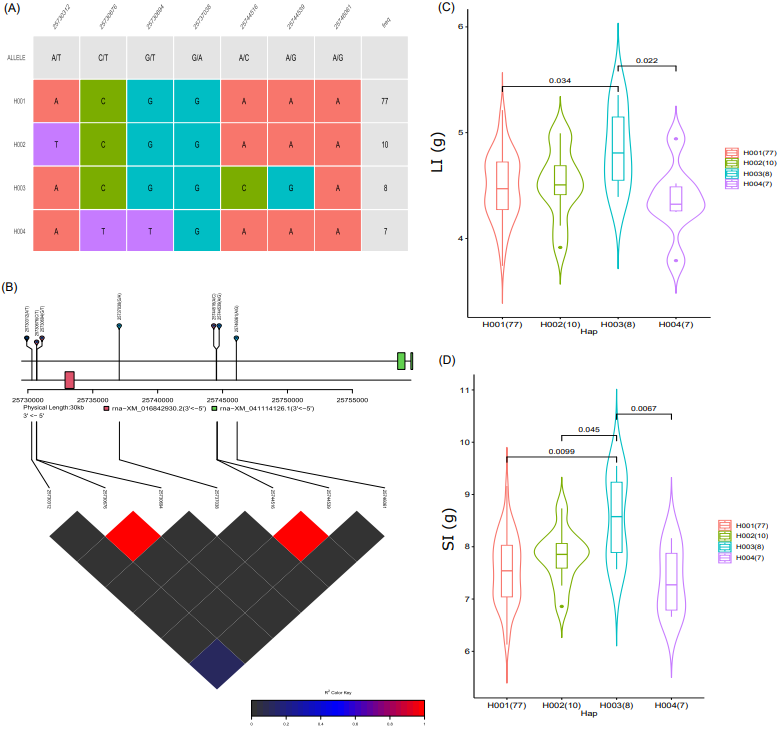


**Supplementary Figure S7.** Haplotype analysis of pleiotropic QTL *qGhSI_LI_A5*, (A) Haplogroups observed in our 117 association panel using seven SNP markers, (B) Genomic location of seven SNP loci and LD based on the pairwise R^2^ values between the SNPs estimated in Chromosome D06. The R^2^ values are indicated using the color bar, (C-D) Phenotypic differences of lint index (LI) g, and seed index (SI) g among the four haplogroups.


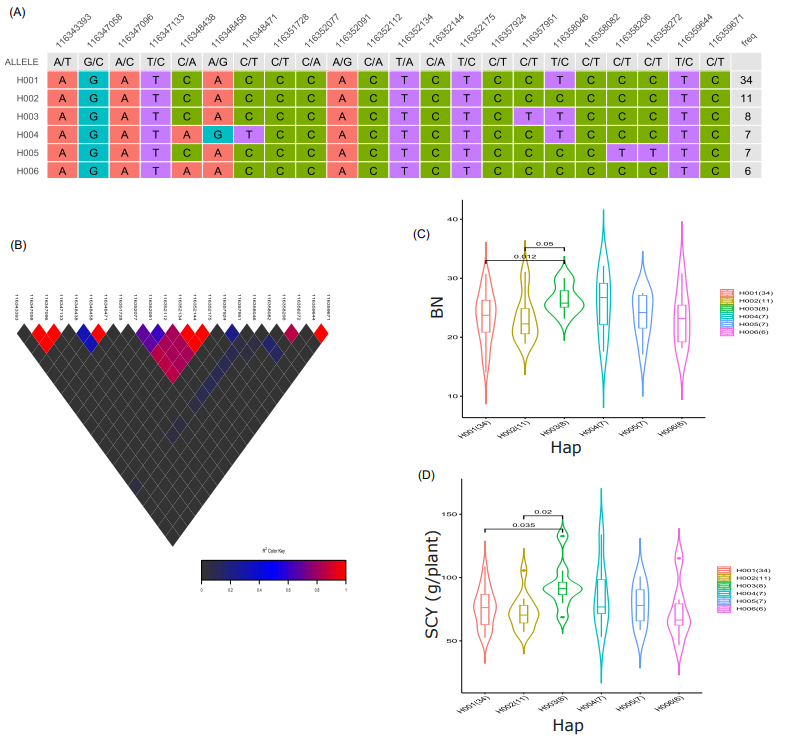


**Supplementary Figure S8.** Haplotype analysis of pleiotropic QTL *qGhBW_SCY_A10*, (A) Haplogroups observed in our 117 association panel using 22 SNP markers, (B) Genomic location of 22 SNP loci and LD based on the pairwise R^2^ values between the SNPs estimated in Chromosome D06. The R^2^ values are indicated using the color bar, (C-D) Phenotypic differences of boll weight (BW) g and seed cotton yield (SCY) g/plant among the six haplogroups.


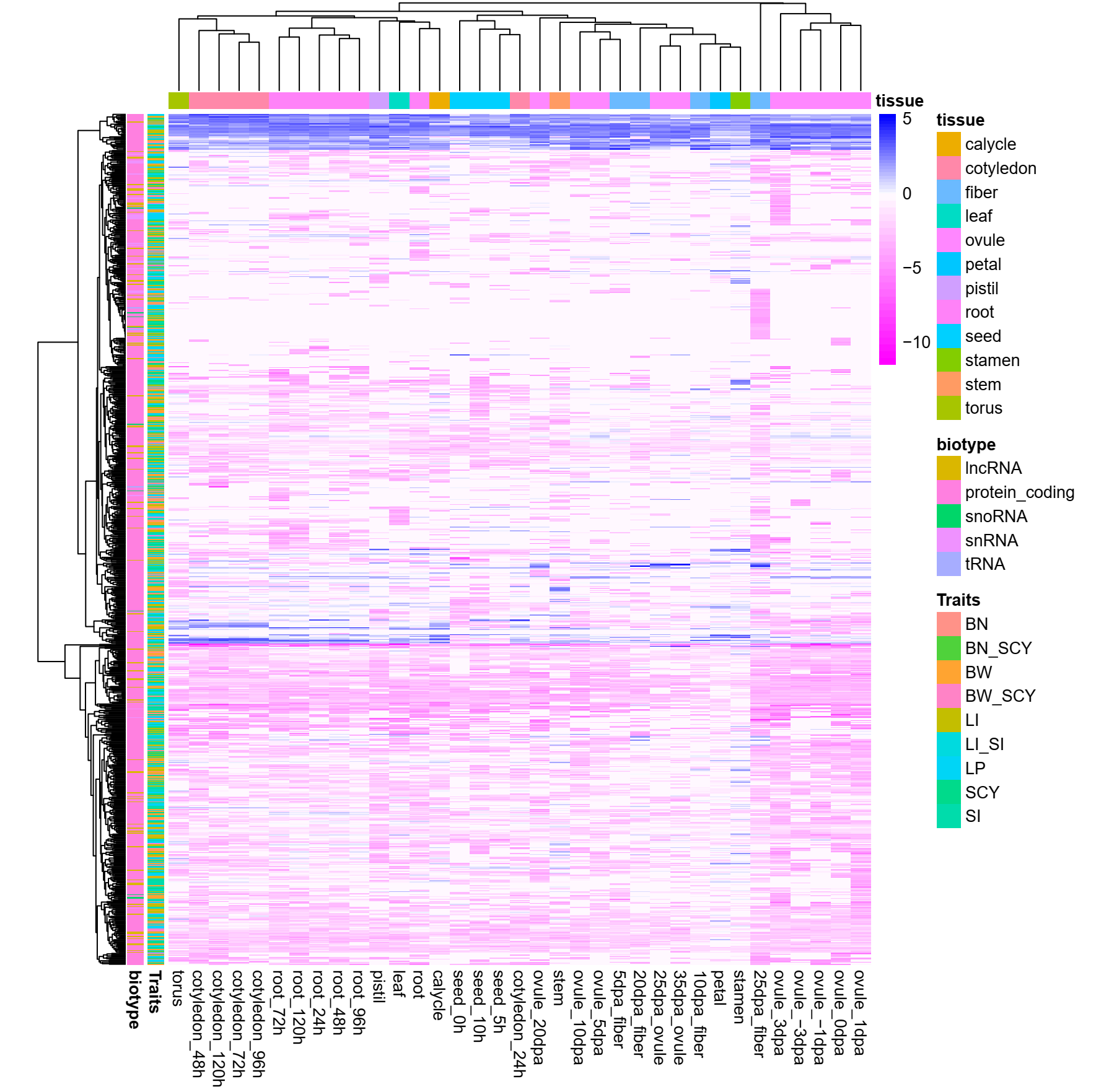


**Figure S9.** Expression pattern of 870 putative genes in all the diverse cotton tissues. The color scale in heatmap represents zFPKM transformed value. Column label is provided for type of tissue and row label is provided for gene biotype, chromosomal location of gene and candidate gene traits
